# Modulation of intrinsic inhibitory checkpoints using nano‐carriers to unleash NK cell activity

**DOI:** 10.15252/emmm.202114073

**Published:** 2021-11-02

**Authors:** Guy Biber, Batel Sabag, Anat Raiff, Aviad Ben‐Shmuel, Abhishek Puthenveetil, Jennifer I C Benichou, Tammir Jubany, Moria Levy, Shiran Killner, Mira Barda‐Saad

**Affiliations:** ^1^ The Mina and Everard Goodman Faculty of Life Sciences Bar‐Ilan University Ramat‐Gan Israel

**Keywords:** Cbl, immunotherapy, nanoparticles, NK cells, SHP‐1, Cancer, Immunology

## Abstract

Natural killer (NK) cells provide a powerful weapon mediating immune defense against viral infections, tumor growth, and metastatic spread. NK cells demonstrate great potential for cancer immunotherapy; they can rapidly and directly kill cancer cells in the absence of MHC‐dependent antigen presentation and can initiate a robust immune response in the tumor microenvironment (TME). Nevertheless, current NK cell‐based immunotherapies have several drawbacks, such as the requirement for *ex vivo* expansion of modified NK cells, and low transduction efficiency. Furthermore, to date, no clinical trial has demonstrated a significant benefit for NK‐based therapies in patients with advanced solid tumors, mainly due to the suppressive TME. To overcome current obstacles in NK cell‐based immunotherapies, we describe here a non‐viral lipid nanoparticle‐based delivery system that encapsulates small interfering RNAs (siRNAs) to gene silence the key intrinsic inhibitory NK cell molecules, SHP‐1, Cbl‐b, and c‐Cbl. The nanoparticles (NPs) target NK cells *in vivo*, silence inhibitory checkpoint signaling molecules, and unleash NK cell activity to eliminate tumors. Thus, the novel NP‐based system developed here may serve as a powerful tool for future NK cell‐based therapeutic approaches.

## Graphical abstract

Natural Killer (NK) cells serve as a first line of immune defense against tumor growth and viral infections. This study demonstrates a nanobiology‐based drug delivery system to enhance NK cytotoxicity by suppressing intracellular inhibitory checkpoints in the tumor microenvironment (TME).

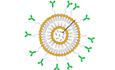

**The paper explained**

**Problem**

Current Natural Killer (NK) cell‐based immunotherapy relies heavily on adoptive transfer and *ex vivo* manufacture of NK cells, which has major limitations to achieve therapeutic impact. Moreover, NK cells express multiple inhibitory checkpoint receptors. Therefore, even if a given receptor is effectively blocked, NK cells may still be inhibited via alternative pathways, compromising the efficiency of this approach. These current limitations call for a novel approach for targeting prevailing intracellular inhibitory signaling cascades shared by multiple surface inhibitory receptors, to unleash NK cells against cancer.

**Results**

In this study, we developed a novel non‐viral lipid nanoparticle‐based delivery system encapsulating small interfering RNAs (siRNAs) targeting three key negative regulatory genes (i) SHP‐1, (ii) Cbl‐b, and (iii) c‐Cbl.

We demonstrate that these nano‐carriers effectively enhance NK cell activity against HLA‐matched cancer cells. These nanoparticles (NPs) also provide an effective *in vivo* delivery system to enhance NK cytotoxicity in the tumor microenvironment (TME).

Targeting NK cells *in vivo* bypasses the need for *ex vivo* isolation of NK cells. Furthermore, this technology provides an innovative and broad therapeutic approach that includes both the active‐modulating compounds and the systemic delivery platform.

**Impact**

The nano‐based delivery system that targets key intracellular inhibitory checkpoints represents a promising immunotherapy for improving NK cells killing activity against cancer in the TME, *in vivo*.

## Introduction

Natural killer (NK) cells are cytolytic effector lymphocytes that participate in the surveillance of stressed, infected, or cancerous cells (Orange *et al*, 2006; Orange, 2008; Vivier *et al*, 2011). NK cells exert their effector functions through secretion of cytolytic granules, which induce target cell apoptosis, and secretion of cytokines, which can activate the adaptive immune response (Vivier *et al*, 2008). Downregulation or lack of major histocompatibility complex (MHC) class‐I molecules increases NK cell activity by diminishing inhibitory signals transduced through inhibitory killer‐cell immunoglobulin‐like receptor (KIR): MHC interactions (Ljunggren *et al*, 2007; Dahlberg *et al*, 2015). Moreover, NK cell responses depend on a balance between activating and inhibitory signaling cascades, derived from activating and inhibiting surface receptors. Common NK cell‐activating receptors include CD16, natural cytotoxicity receptors (NCRs) NKp46, NKp44, NKp30, and NKp80, 2B4, and natural killer group 2 member D (NKG2D) (Moretta *et al*, 2001; Welte *et al*, 2006; Amand *et al*, 2017).

The critical human inhibitory NK cell receptors, KIRs, have long cytoplasmic tails (KIR‐L) containing two immunoreceptor tyrosine‐based inhibitory motifs (ITIMs). Each KIR recognizes a subgroup of human leukocyte antigen (HLA) class I allotypes and displays two (KIR2DL) or three (KIR3DL) extracellular Ig‐domains, conferring specificity for HLA‐C or HLA‐A/B allotypes, respectively (Thielens *et al*, 2012). Engagement of KIR receptors with cognate MHC ligands induces the recruitment of Src homology 2 (SH2) domain‐containing protein tyrosine phosphatase–1 (SHP‐1) to the KIR ITIM domains (Poole *et al*, 2005). SHP‐1 was previously shown to inhibit NK cell activity by dephosphorylating the Guanine nucleotide exchange factor, VAV1, and as we previously demonstrated, the Linker for activation of T cells (LAT), phospholipase C‐γ1 (PLC‐γ1), and PLC‐γ2 (Stebbins *et al*, 2003; Campbell, 2016b; Matalon *et al*, 2016). In addition to SHP‐1, the E3 ubiquitin ligases Cbl‐b and c‐Cbl serve as key negative regulators of NK cell activity (Paolino *et al*, 2014).

We previously showed that during NK cell inhibition, LAT is ubiquitylated by Cbl‐b and c‐Cbl (Campbell & Bennett, 2016; Matalon *et al*, 2016), leading to LAT degradation and thereby abolishing NK cell activation and cytotoxicity. Furthermore, a previous study in NK cells showed that genetic deletion of the E3 ubiquitin ligase, Cbl‐b, or targeted inactivation of its E3 ligase activity activates NK cells to spontaneously reject metastatic tumors (Paolino *et al*, 2014). Furthermore, c‐Cbl inhibits VAV1, resulting in the suppression of NF‐κB‐driven cytotoxic programs (Kwon *et al*, 2017). Thus, it is evident that both SHP‐1 and Cbls play critical roles in dictating the NK cell activation threshold, and are therefore attractive targets for NK cell‐based immunotherapy.

Cancer immunotherapy is designed to enhance immune cell activity against cancer cells while leaving normal cells unharmed (Wang *et al*, 2016; Ott *et al*, 2017). Most immunotherapeutic approaches focus on the adaptive immune system for tumor immune surveillance, although increasing evidence supports a significant role for innate immune effector cells, such as NK cells, in the elimination of tumors (Marcus *et al*, 2014). NK cell‐based immunotherapy offers significant advantages over current immune‐based treatments, including the ability of NK cells to kill tumor cells directly without relying on recognition of specific tumor antigens, and reduced non‐specific toxicity compared to T cell‐based therapies (Vivier *et al*, 2011; Paolino *et al*, 2014; Guillerey *et al*, 2016; Esin *et al*, 2017; Martín‐Antonio *et al*, 2017; Bugide *et al*, 2018). NK cells can also complement T‐cell immune surveillance by targeting MHC‐I‐deficient tumors, which decrease β2‐microglobulin expression to escape T‐cell activity (Rodríguez, 2017). Furthermore, NK cells can initiate cross‐talk with dendritic cells and T cells in the tumor microenvironment (TME), resulting in the recruitment of potent adaptive anti‐tumor responses (Ferlazzo *et al*, 2014). Current NK cell‐based treatments rely heavily on adoptive transfer; however, *ex vivo* manufacture of NK cell‐based therapeutics has major limitations, including the need for extensive *ex vivo* expansion procedures subject to a high risk of contamination, lack of sufficient NK cell numbers to attain therapeutic impact, and the reduction of the NK cell cytolytic phenotype (Davies *et al*, 2014; Hu *et al*, 2019). Furthermore, transduction protocols for the generation of chimeric antigen receptor (CAR) expressing NK cells may cause undesired genomic effects, including oncogenic transformation (Hu *et al*, 2018). Therefore, there is increasing interest in developing immunotherapies that harness NK cells *in vivo*.

In contrast to adoptive cell transfer, immune checkpoint inhibition (ICI) therapy aims to block immune checkpoint molecules such as PD‐1 and CLTA‐4 or their ligands, to unleash the immune response *in vivo* (Parry *et al*, 2005; Conniot *et al*, 2019). For NK cells, these inhibitory checkpoints include cell surface receptors such as KIRs and CD94/NKG2A, and may also include PD‐1, TIM‐3, TIGIT, CEACAM1, CD96, and LAG‐3, expressed on both NK and T cells (Topalian *et al*, 2015). Recent evidence demonstrates that ICI targeting NK cell surface receptors could be a potential therapeutic avenue for cancer immunotherapy (Souza‐Fonseca‐Guimaraes *et al*, 2019; Sun *et al*, 2019), yet ICI is beneficial only for a subset of patients and cancer types (Seidel *et al*, 2018). Often, immune editing of the tumor niche provides resistance to conventional checkpoint blockade (Jenkins *et al*, 2018). In the context of NK cell immune surveillance, tumors can often upregulate particular HLA molecules such as HLA‐E/G to repress NK cell activity (Freund‐Brown *et al*, 2018). Moreover, the suppressive cellular milieu of the TME (e.g., stromal cells, fibroblasts, regulatory T cells, and myeloid suppressor cells), in addition to inhibitory cytokines (e.g., TGF‐β, IL‐10), can induce T/NK cell dysfunction (Vasievich *et al*, 2011). A major caveat limiting the efficacy of immune checkpoint blockade therapy is the fact that multiple inhibitory checkpoint receptors are expressed on the surface of T and NK cells; therefore, even if a given receptor is effectively blocked, NK cells may still be inhibited via alternative pathways, compromising the efficiency of this approach. Simultaneously blocking every inhibitory checkpoint receptor to overcome exhaustion in the TME is not a feasible option. These current limitations in immunotherapy call for a novel approach for targeting prevailing inhibitory signaling cascades shared by multiple inhibitory receptors, to unleash NK cells against cancer.

Here, we engineered a non‐viral delivery system, lipid‐based nano‐carriers encapsulating small interfering RNAs (siRNAs), to target common intrinsic inhibitory NK cell signaling pathways. The nano‐carriers encapsulate siRNAs targeting three genes critical for suppression of NK cell activity, namely, SHP‐1, Cbl‐b, and c‐Cbl. We employed a target cell system in which NK cell activity is repressed through the common immune‐editing pathway of high expression of the ligands for the KIR inhibitory checkpoint receptor. We demonstrate that nano‐carriers effectively and safely silence SHP‐1 and Cbls in NK cells *in vitro* and *in vivo*, unleashing NK cell activity against HLA‐matched cancer cells, and prolong survival in humanized murine models. Our study demonstrates that nanoparticles (NPs) encapsulating small molecules provide an effective systemic *in vivo* delivery strategy to enhance NK cytotoxicity in the TME.

## Results

### Gene silencing of SHP‐1 and Cbls enhances NK cell activity

**Figure EV1:**
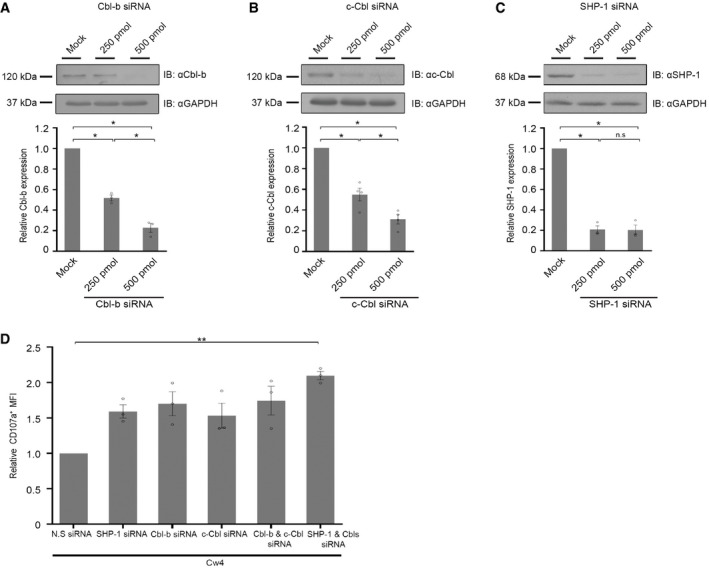
Gene silencing of SHP‐1 and Cbls enhances NK cell function YTS‐2DL1 cells were either mock‐transfected or transfected with 250 or 500 pmol of (A) Cbl‐b siRNA, (B) c‐Cbl siRNA, or (C) SHP‐1 siRNA using Amaxa electroporation. After 48 h, cells were lysed, and the nitrocellulose membranes were blotted with anti‐Cbl‐b, anti‐c‐Cbl, or anti‐SHP‐1 antibodies. GAPDH served as a loading control. Densitometric analysis of the bands was performed using ImageJ and normalized to the GAPDH densitometry values. Relative expression of the three proteins compared to the mock control group is presented within the graph. Analysis by ImageJ densitometry revealed a decrease of Cbl‐b siRNA: 48 ± 3% and 77 ± 4%, *P* ≤ 0.03 for Cbl‐b, 45 ± 6% and 68 ± 4%, *P* ≤ 0.03 for c‐Cbl, and 79 ± 3% and 79 ± 5%, *P* ≤ 0.03 for SHP‐1 following siRNA gene silencing concentrations of 250 pmol and 500 pmol, respectively (shown in bar graphs underneath each blot). The data represent three independent experiments (*n* = 3). Data are shown as mean ± SEM. *P* values were calculated vs mock‐treated control cells by one‐sample *t*‐tests and independent *t*‐test. *P* values are indicated by asterisks. *P ≤ 0.05.YTS KIR2DL1 cells were gene silenced for either SHP‐1, Cbl‐b, c‐Cbl or a combination of both Cbls proteins or of Cbls and Shp‐1. NK cells treated with N.S siRNA served as control. After 48 h, YTS KIR2DL1 were incubated with 721.221 Cw4 target cells for 2 h and analyzed by flow cytometry to determine the expression of CD107a. Expression of CD107a was compared by mean fluorescence intensity (MFI). Relative expression of CD107a MFI was normalized to the mock‐transfected sample following Cw4 incubation. The data represent three independent experiments (*n* = 3). Data are shown as mean ± SEM. *P* values were calculated by one‐sample *t*‐test and are indicated by asterisks. **P ≤ 0.001. Data information: Exact *P* values are shown in Appendix Table S1.

**Figure 1:**
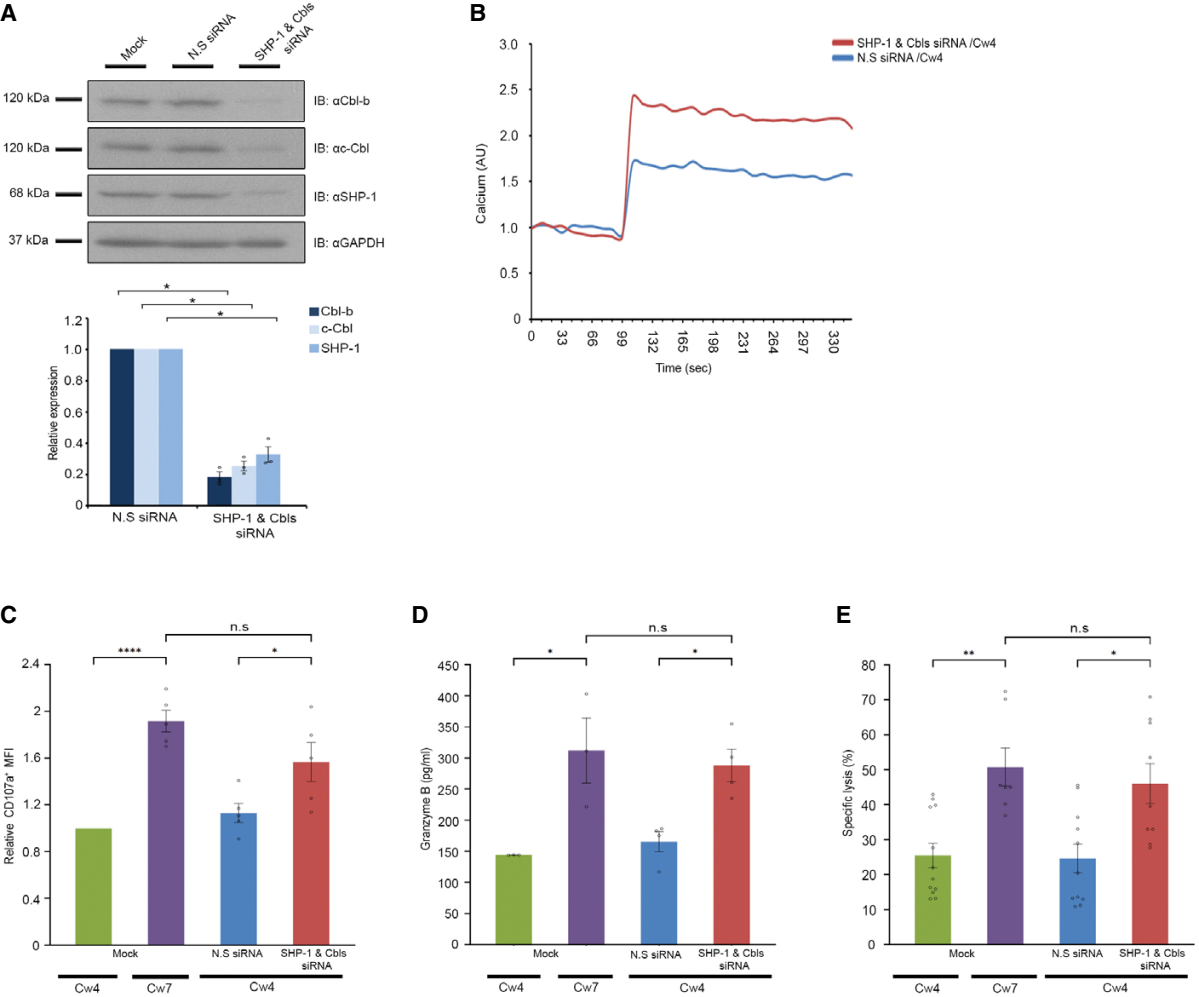
Inhibition of Cbl‐b, c‐Cbl, and SHP‐1 enhance NK cell function YTS KIR2DL1 cells were mock‐transfected or transfected with N.S siRNA or Cbl‐b siRNA, c‐Cbl siRNA and SHP‐1 siRNA, using Amaxa electroporation. After 48 h, cells were lysed and subjected to Western blot with anti‐Cbl‐b, anti‐c‐Cbl or anti‐SHP‐1 antibodies. Analysis by ImageJ densitometry is summarized in the graph below. Data are means ± SEM of three independent experiments (*n* = 3). *P* values were calculated by one‐sample *t*‐test and are indicated by asterisks *P < 0.05.YTS KIR2DL1 cells were transfected with either N.S siRNA (blue curve) or with SHP‐1 and Cbls siRNA (red curve). After 48 h, cells were loaded with calcium‐sensitive Fluo‐3‐AM and analyzed for basal intracellular calcium levels for 100 s. The NK cells were then mixed with 721.221 Cw4 target cells at 37°C and monitoring of calcium levels was continued. The data shown are representative of three independent experiments.YTS KIR2DL1 cells were incubated with either 721.221 Cw4 or Cw7 target cells for 2 h and analyzed by flow cytometry to determine the expression of CD107a. Expression of CD107a was measured by mean fluorescence intensity (MFI). Relative expression of CD107a MFI was normalized to the mock‐transfected sample following Cw4 incubation. Data are means ± SEM of five independent experiments (*n* = 5). *P* values were calculated by one‐sample *t*‐tests and one‐way ANOVA following Tukey's *post hoc* analysis are indicated by asterisks *P < 0.05, ****P < 0.0001.Granzyme release. YTS KIR2DL1 cells were mock‐transfected or transfected with N.S siRNA or SHP‐1 and Cbls siRNA. After 48 h, cells were incubated with either 721.221 Cw4 or Cw7 target cells, the supernatant was then collected and granzyme B levels were evaluated using ELISA sandwich assay. The levels of granzyme B were quantified using standard recombinant granzyme B concentrations. Data are means ± SEM of eight independent experiments (*n* = 8); *P* values were calculated by one‐way ANOVA following Tukey's *post hoc* analysis and are indicated by asterisks *P < 0.05.Killing assay. YTS KIR2DL1 cells were mock‐transfected or transfected with N.S siRNA or SHP‐1 and Cbls siRNA. After 48 h, cells were incubated with [^35^S] Met‐loaded 721.221 Cw4 or Cw7 target cells. After 5 h of co‐culture, the supernatant was collected, and the radioactive signal was measured. Data are means ± SEM of three independent experiments (*n*= 3). *P* values were calculated by one‐way ANOVA following Tukey's *post hoc* analysis and are indicated by asterisks *P < 0.05, **P < 0.01. Data information: Exact *P* values are shown in Appendix Table S1. Source data are available online for this figure.

To suppress the key inhibitors of NK cell cytotoxicity, we designed siRNAs targeting Cbls and SHP‐1. For this purpose, YTS KIR2DL1 (henceforth referred to as YTS‐2DL1) cells were transfected with 250 or 500 pmol of Cbl‐b (Fig EV1A), c‐Cbl (Fig EV1B), or SHP‐1 (Fig EV1C) siRNA and monitored for gene silencing efficiency after 48 h. A significant decrease in all the three proteins was detected relative to non‐specific (N.S) siRNA control. More efficient gene silencing was obtained by using 500 pmol of siRNA for Cbl‐b and c‐Cbl (Cbl‐b siRNA: 250 pmol vs and 500 pmol *P* = 0.05; c‐Cbl siRNA: 250 pmol vs and 500 pmol *P* = 0.03), but the efficiency of SHP‐1 gene silencing was not affected by the siRNA concentration. A concentration of 250 pmol was selected for use in further experiments for each of these three proteins whenever they were targeted without being encapsulated in NPs. To determine the effect of our designed SHP‐1 and Cbls siRNAs on the NK cell activation threshold, we used siRNAs to target each of the proteins individually or in combination. In an attempt to combine the three siRNAs in lipid‐based NPs and to avoid potential off‐target effects and cell damage (Jackson *et al*, 2010), we decided to use 250 pmol/each siRNA. YTS‐2DL1 cells were transfected with Cbl‐b, c‐Cbl, and SHP‐1 siRNAs. Gene silencing was determined by Western blot analysis after 48 h. A significant decrease was observed in the expression of Cbl‐b, c‐Cbl, and SHP‐1 relative to the control, non‐specific siRNA‐treated cells (Fig 1A).

Next, we used functional assays to determine the effect of SHP‐1 and Cbls gene silencing in enhancing NK cell activation following inhibitory interactions. To determine the impact of SHP‐1 and Cbl repression of NK cell activity, we simulated a common immune‐editing pathway that inhibits NK cells: high surface expression of HLA ligands for KIR receptors. To this end, YTS‐2DL1 cells were incubated with 721.221 target cells that overexpress the HLA–Cw4, a cognate MHC Class I ligand for the KIR2DL1 inhibitory receptor (221‐Cw4 cells), which induces NK cell inhibition, or with 721.221 targets expressing the irrelevant HLA‐Cw7 (221‐Cw7 cells) molecule. This allotype is not recognized by the KIR2DL1 receptor and therefore target cells expressing it are recognized and lysed by NK cells. As can be seen in Fig EV1D, NK cell degranulation (LAMP1, CD107a expression) was higher in YTS‐2DL1 that were transfected with a combination of SHP‐1 and Cbl siRNAs and interacted with 221‐Cw4 cells, relative to the levels observed in YTS‐2DL1 transfected with N.S siRNA, or siRNA targeting SHP‐1, Cbl‐b, and c‐Cbl, individually (Fig EV1D). These results demonstrate that Cbl‐b, c‐Cbl, and SHP‐1 tune the NK cell activation threshold. Therefore, these intrinsic inhibitory checkpoint molecules are attractive targets for increasing NK cell function.

### Combined inhibition of SHP‐1 and Cbls controls the balance of NK cell activation

**Figure EV2:**
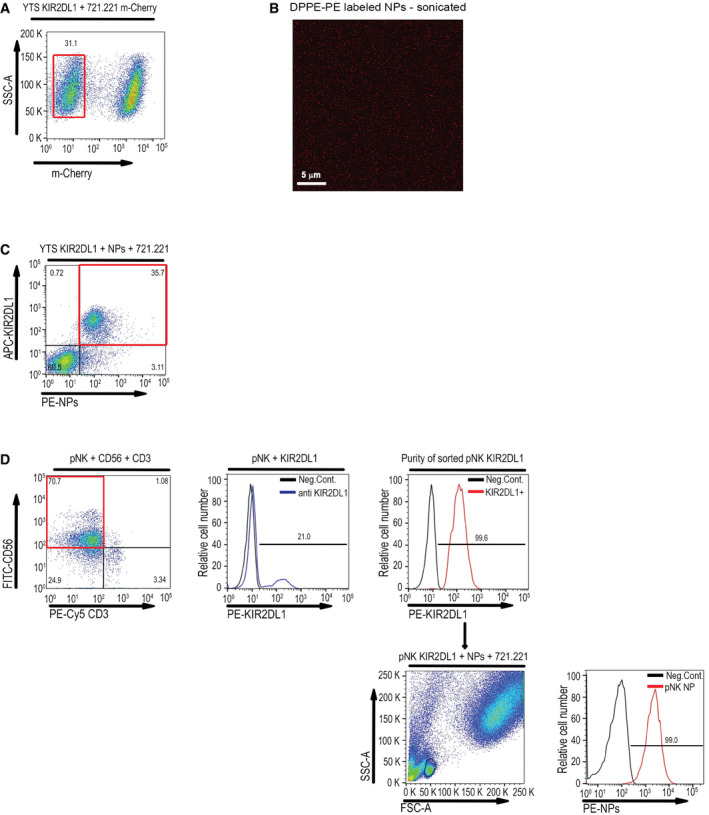
Gating strategies for the identification of NK cells Gating strategy to distinguish between YTS‐2DL1 cells and 221‐Cw4/7‐expressing mCherry cells, using side scatter (SSC) and mCherry. Cells that were negative for mCherry were analyzed for CD107a expression.Imaging of fluorescently labeled NPs using confocal microscopy following sonication. The data shown are representative of three independent experiments.Gating strategy to define YTS‐2DL1 incorporated NKp46 antibody‐coated NPs population, using anti‐KIR2DL1 to define NK cells. Cells that were positive for both rhodamine‐labeled NPs and KIR2DL1 antibody staining were analyzed for CD107a expression.Primary NK cells were stained with PE‐Cy5‐CD3 and FITC‐CD56 antibodies followed by staining with PE‐KIR2DL1/S1. The pNK‐expressing KIR2DL1 subset was then enriched by FACS sorting according to the PE signal. This subset was then incubated with target cells. Gating strategy to define the pNK‐KIR2DL1^+^ using forward scatter (FSC) and side scatter (SSC). Cells that were positive for rhodamine‐labeled NPs were analyzed for CD107a expression.

After validating an optimal concentration for efficient combined knockdown of Cbls and SHP‐1 (Fig 1A), we next determined whether gene silencing of these intrinsic inhibitory checkpoints reduces the NK cell activation threshold and increases NK cell functional activities. Accordingly, YTS‐2DL1 were incubated with 221‐Cw4 target cells, resulting in NK cell inhibition. As seen in Fig 1B, intracellular calcium levels following inhibitory interactions with 221‐Cw4 target cells were markedly elevated in YTS‐2DL1 cells gene silenced for SHP‐1 and Cbls, relative to the levels observed in YTS‐2DL1 transfected with N.S siRNA. In addition, YTS‐2DL1 transfected with SHP‐1 and Cbls siRNA exhibited a significant increase in their ability to secrete cytolytic granules following inhibitory interactions (721‐Cw4 mCherry) compared to mock‐transfected cells or cells transfected with NS siRNA that were subjected to inhibitory interactions (1.83 ± 0.3 relative CD107a levels, *P* ≤ 0.04; Figs 1C and EV2A). The increase in degranulation of NK cells silenced for SHP‐1 and Cbls resembled the degranulation levels during an activating NK cell response (SHP‐1 and Cbls siRNA/Cw4 1.83 ± 0.3; Mock/Cw7 1.72 ± 0.1, *P* = 0.1). These results were also verified by measuring granzyme B release (Fig 1D). YTS‐2DL1 cells gene silenced for SHP‐1 and Cbls that were incubated with inhibitory 221‐Cw4 cells exhibited a significant increase of granzyme B secretion compared to YTS‐2DL1 pretreated with N.S siRNA (SHP‐1 and Cbls siRNA/Cw4 287.9 ± 26.1 pg/ml; N.S siRNA/Cw4 165.4 ± 16.3 pg/ml, *P* = 0.003). Finally, we established the role of SHP‐1 and Cbls in downregulating NK cell cytotoxicity by direct measurement of NK cell killing of target cells. To this end, we utilized a standard radioactive [^35^S] Met release killing assay (Fig 1E). NK cells that were gene silenced for SHP‐1 and Cbls and incubated with 221‐Cw4 cells demonstrated superior killing of target cells relative to N.S siRNA‐transfected cells (SHP‐1 and Cbls siRNA/Cw4 46.02 ± 5.69%; N.S siRNA/Cw4 24.67 ± 4.1%, *P* = 0.01). Interestingly, NK cells that were gene silenced for SHP‐1 and Cbls and incubated with inhibitory 221‐Cw4 cells showed no significant difference relative to NK cells incubated with activating 221‐Cw7 cells (Mock/Cw7 50.76 ± 5.44%*,P* = 0.91). Altogether, these data demonstrate that Cbl‐b, c‐Cbl, and SHP‐1 serve as negative key checkpoints suppressing NK cell function, and thus, inhibition of these key intrinsic checkpoints overcomes inhibitory signaling.

### Lipid‐based nanoparticles for SHP‐1 and Cbl siRNA delivery

Liposomal NPs are pharmaceutically proven delivery vehicles that can encapsulate a therapeutic agent, and can also display ligands that target cell surface receptors (Torchilin, 2005). Leukocytes are challenging targets for delivery due to their dispersion within the body, making it difficult to successfully localize or passively deliver molecules via systemic administration (Bitko *et al*, 2005). To circumvent these obstacles, we engineered NK cell‐targeting NPs. Liposome‐based NPs are efficient in transporting RNAi molecules for the treatment of different malignancies (Peer, 2013). These particles have a lipid bilayer with an internal hydrophilic space—maintaining the payload segregated from the bloodstream. This bilayer formation protects the encapsulated particle content from being degraded by immune cells and enzymes in the bloodstream. Furthermore, the external liposome layer, which enables the coating of its polymeric components, does not induce an immune system response and is not toxic, preventing unwanted side effects (Daka *et al*, 2012).

**Figure 2:**
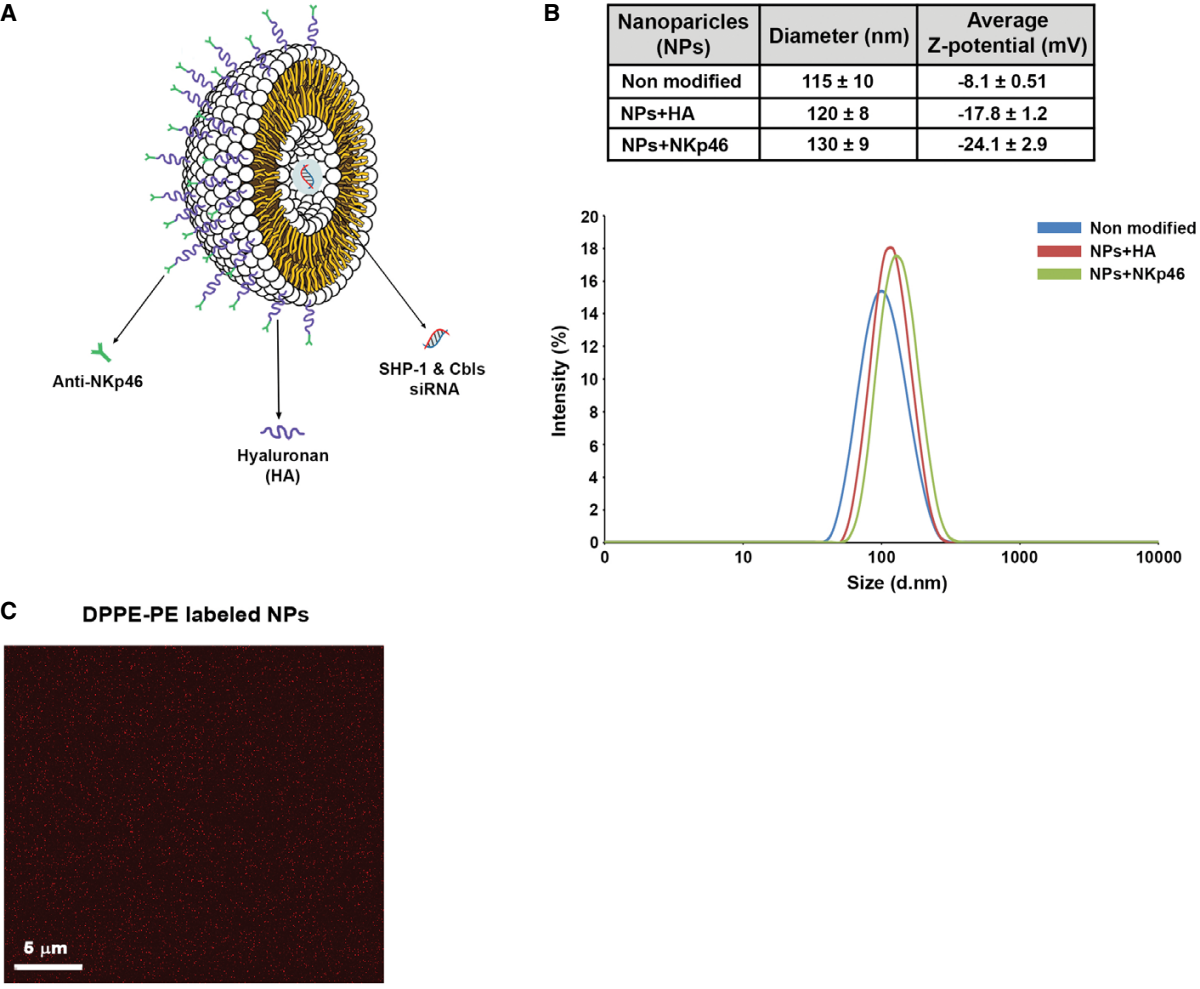
Structure and characterization of liposomal NPs Schematic presentation of liposomal NP layers and composition.Characterization of the diameter and zeta potential (Z‐potential) with the addition of each layer. Graph below presenting NP size distribution.Imaging of fluorescently labeled NPs using confocal microscopy. The data shown are representative of three independent experiments.

To enable the targeting of NK cells, NPs were coated with a monoclonal anti‐NKp46 antibody (Yossef *et al*, 2015), which can recognize NK cells via their NKp46 activating receptor (Fig 2A). This receptor serves as a useful marker for NK cell targeting, since it is expressed on all mature NK cells (Glasner *et al*, 2012). Furthermore, recent work demonstrated that NKp46 expression in tumor‐associated NK cells of multiple cancer types is relatively stable compared to that of other activating NK receptors in cancers, such as CD16, NKG2D, and NKp30, which can undergo proteolytic cleavage (Zingoni *et al*, 2016; Gauthier *et al*, 2019). Therefore, NKp46 is a prominent marker for NK cells and provides a stable target for NP delivery. The size and the charge of the NPs during the preparatory stages were assessed using dynamic light scattering (DLS; Fig 2B). To monitor NP's distribution and to verify they do not form aggregates, DPPE labeled with rhodamine red (DPPE‐PE) was incorporated into the lipid mixture, allowing NP visualization as demonstrated by confocal microscopy (Figs 2C and EV2B).

### Antibody‐coated lipid‐based nanoparticles target NK cells via the NKp46 surface receptor

**Figure 3:**
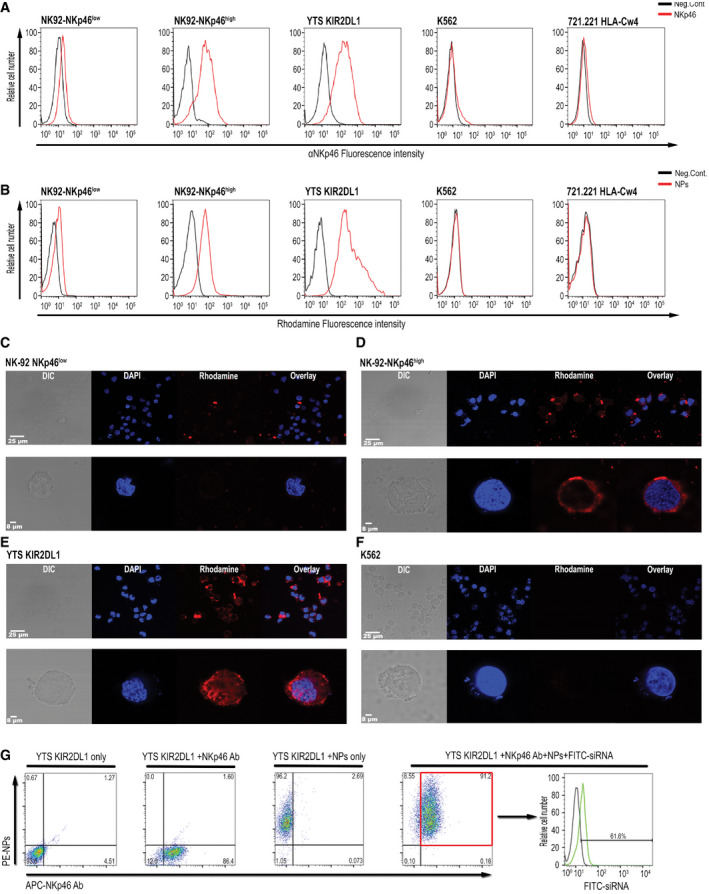
Specific and efficient siRNA delivery to NK cells using NKp46 antibody‐coated NPs ANK92‐NKp46^low^, NK92‐NKp46^high^, YTS KIR2DL1, K562, and 721.221 HLA‐Cw4 cells were stained with anti‐NKp46 monoclonal antibody followed by staining with Alexa568‐Fluor goat anti‐mouse IgG secondary antibody. Cells were then analyzed using flow cytometry.BNK92‐NKp46^low^, NK92‐NKp46^+^, YTS KIR2DL1, K562, and 721.221 HLA‐Cw4 cells were incubated with 50 µg rhodamine‐labeled NPs and analyzed using flow cytometry to confirm the internalization of the coated NPs in a NKp46‐specific manner.C–F(C) NK92‐NKp46^low^, (D) NK92‐NKp46^high^, (E) YTS KIR2DL1, and (F) K562 cells were incubated with 50 µg rhodamine‐labeled NPs and analyzed using confocal microscopy. Upper panel shows wide field images. Lower panel shows single‐cell images. The data shown are representative of three independent experiments (*n* = 3).GYTS KIR2DL1 were incubated with NKp46 antibody‐coated NPs loaded with FITC‐labeled siRNA. After 24 h, the cells were stained with anti‐NKp46 monoclonal antibody followed by staining with Alexa568‐Fluor goat anti‐mouse IgG secondary antibody. Then, the cells were analyzed by flow cytometry to determine the incorporation of the rhodamine‐labeled NPs loaded with FITC‐siRNA and NKp46 antibody staining. Cells that were positive for both rhodamine‐labeled NPs and NKp46 antibody staining were analyzed for the presence of fluorescent FITC‐siRNA.

The NPs were coated with antibodies against the activating receptor, NKp46. Anti‐NKp46 monoclonal antibodies, which were demonstrated to have no effect on either NK activation or their function, were produced and purified from hybridoma supernatant (Shemer‐Avni *et al*, 2017). These antibodies were evaluated for their specificity and ability to recognize the NKp46 receptor expressed by NK cells. Initially, we tested antibody binding to the NK92 cell line clone that naturally expresses low levels of NKp46 (NK92‐NKp46^low^) and an NK92 clone that expresses high levels of NKp46 (NK92‐NKp46^high^) (Shemer‐Avni *et al*, 2017). In addition, we examined the expression of NKp46 in YTS‐2DL1 and 221‐Cw4 cells, which served as an *in vitro* model in our previous experiments. K562, a chronic myeloid leukemia (CML) cell line that does not express NKp46, was used as a negative control. As can be seen in Fig 3A, we detected high staining of NKp46 in NK92‐NKp46^high^ cells and only weak staining in the NK92‐NKp46^low^ cells. In addition, the YTS‐2DL1 cell line also exhibited high expression of NKp46. As expected, no NKp46 expression was detected in K562 or 221‐Cw4 cells.

We then validated the specificity and uptake of NKp46 antibody‐coated NPs by these same cell lines. Cells were incubated with rhodamine‐labeled NPs for 24 h, and analyzed by flow cytometry. As seen in Fig 3B, NK92‐NKp46^high^ cells contained high levels of the NPs, while only minor uptake was observed in NK92‐NKp46^low^ cells. As expected, the YTS‐2DL1 cell line showed high uptake of the NPs, which is consistent with their NKp46 expression. K562 cells and 221‐Cw4 cells did not show any integration of the NPs. These results demonstrate that the NKp46 antibody‐coated NPs undergo internalization in NK cells with high expression of the NKp46 receptor.

The uptake and internalization of the NKp46 antibody‐coated NPs were also confirmed using confocal microscopy. As seen in Fig 3C, while NK92‐NKp46^low^ showed low internalization of the NPs, we observed a robust presence of the NPs in NK92 NKp46^high^ and YTS‐2DL1 cells (Fig 3D and E). We could not detect any NP internalization in K562 cells (Fig 3F). Finally, we wished to determine the efficiency of siRNA entrapment. For this purpose, YTS‐2DL1 were incubated with NKp46‐NPs loaded with FITC‐NS siRNA and stained with an anti‐NKp46 antibody. As can be seen in Fig 3G, a high percentage of YTS‐2DL1 cells demonstrated NKp46 staining and exhibited incorporation of NKp46 antibody‐coated NPs (91.2%). The YTS‐2DL1 cell population stained for both NKp46 and NPs was subsequently analyzed by flow cytometry and demonstrated high efficiency of siRNA uptake (61.6%).

### NKp46 antibody‐coated NPs downregulate the expression of the intrinsic checkpoint molecules, SHP‐1 and Cbls to unleash NK cell activation

**Figure 4:**
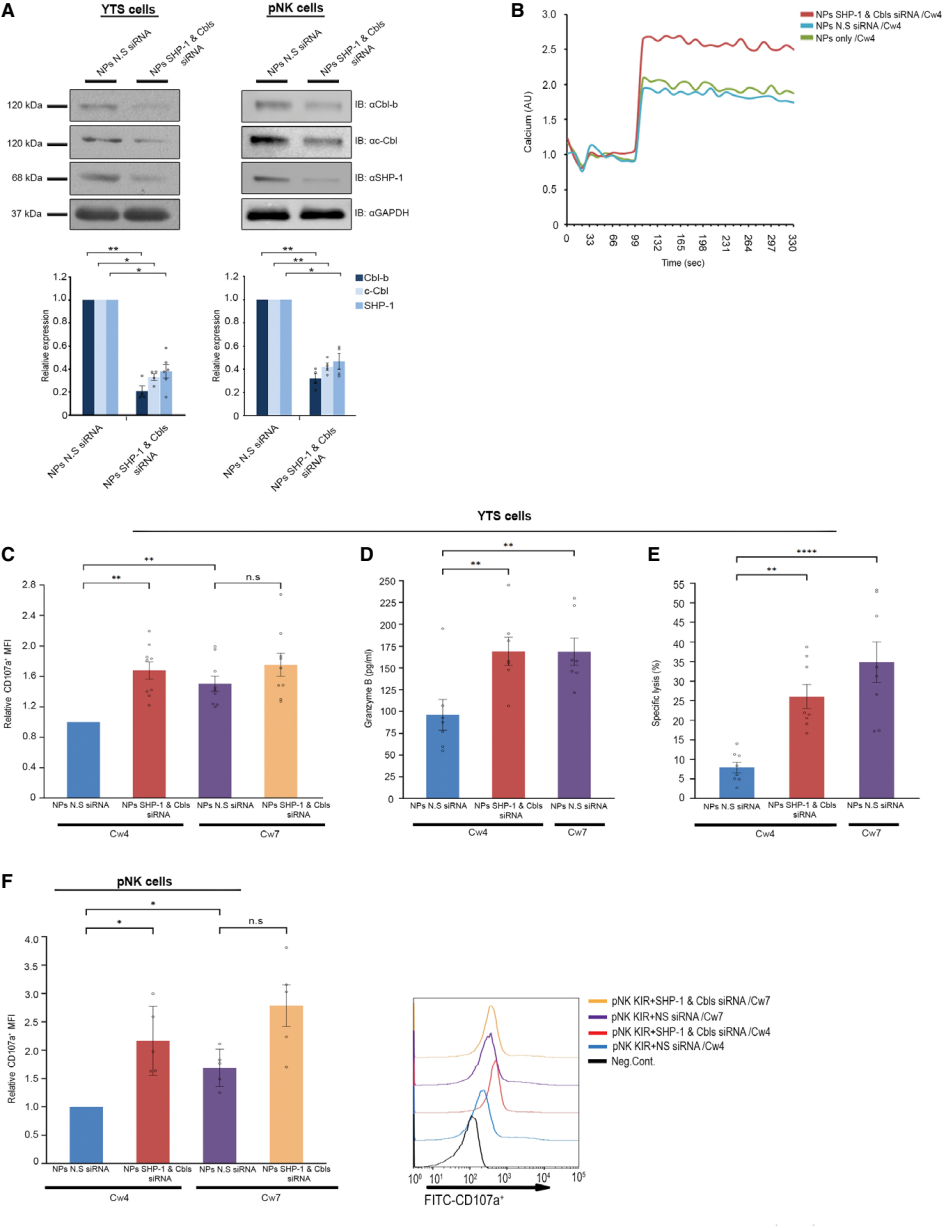
NKp46 antibody‐coated NPs loaded with SHP‐1 and Cbls siRNA enhance NK cell function following inhibitory interactions YTS KIR2DL1 and primary NK cells were incubated with NPs loaded with Cbl‐b, c‐Cbl and SHP‐1 siRNA, or NPs loaded with 1,500 pmol N.S siRNA. Following 48 h of incubation, cells were lysed, and the membrane was blotted with anti‐Cbl‐b, anti‐c‐Cbl, or anti‐SHP‐1 antibodies. GAPDH served as loading control. Densitometric analysis of the bands was performed using ImageJ and normalized to the GAPDH densitometry values. Analysis by ImageJ densitometry is summarized in the graph below. Data are means ± SEM of four independent experiments (*n* = 4). *P* values were calculated by one‐sample *t*‐tests and are indicated by asterisks. *P < 0.05, **P < 0.01.YTS KIR2DL1 cells were incubated with SHP‐1 and Cbls siRNAs‐loaded NPs (red curve), with NS siRNA‐loaded NPs (green curve) or with empty NPs (blue curve). After 48 h, cells were loaded with calcium‐sensitive Fluo‐3‐AM and analyzed for basal intracellular calcium levels for 1 min. The NK cells were then mixed with 721.221 Cw4 target cells at 37°C, and monitoring of calcium levels was continued. The data shown are representative of three independent experiments.YTS KIR2DL1 cells were pretreated either SHP‐1 and Cbls siRNA or N.S siRNA‐loaded NPs. After 48 h, cells were incubated with either 721.221 Cw4 or Cw7 target cells for 2 h and analyzed by flow cytometry to determine the expression of CD107a. Expression of CD107a was quantitated by mean fluorescence intensity (MFI). Data are means ± SEM of nine independent experiments (*n* = 9). *P* values were calculated by one‐sample *t*‐tests and one‐way ANOVA following Tukey's *post hoc* analysis and are indicated by asterisks. **P < 0.01.YTS KIR2DL1 cells were treated with SHP‐1 and Cbls siRNAs‐loaded NPs or N.S siRNA‐loaded NPs. After 48 h, cells were incubated with either 721.221 Cw4 or Cw7 target cells, the supernatant was then collected, and granzyme B levels were evaluated using ELISA sandwich assay. The levels of granzyme B were quantified based on a standard curve. Data are means ± SEM of five independent experiments (*n* = 5). *P* values were calculated by one‐way ANOVA following Tukey's *post hoc* analysis and are indicated by asterisks. **P < 0.01.YTS KIR2DL1 cells were pretreated with either SHP‐1 and Cbls siRNA‐loaded NPs, or N.S siRNA‐loaded NPs. After 48 h, cells were incubated with [^35^S] Met‐loaded 721.221 Cw4 or Cw7 target cells. After co‐culture for 5 h, the supernatant was collected, and radioactive signal was measured. Data are means ± SEM of seven independent experiments (*n* = 7). *P* values were calculated by one‐way ANOVA following Tukey's *post hoc* analysis and are indicated by asterisks **P < 0.01 ****P < 0.0001.pNK cells were pretreated with either SHP‐1 and Cbls siRNA‐loaded NPs or N.S siRNA‐loaded NPs. After 48 h, cells were incubated with either 721.221 Cw4 or Cw7 target cells for 2 h and analyzed by flow cytometry to determine the expression of CD107a. Expression of CD107a was quantitated by mean fluorescence intensity (MFI). The data represent five independent experiments (*n* = 5). Data are shown as mean ± SEM. *P* values were calculated by one‐sample *t*‐tests and one‐way ANOVA following Tukey's *post hoc* analysis and are indicated by asterisks. *P ≤ 0.05. Data information: Exact *P* values are shown in Appendix Table S1. Source data are available online for this figure.

The ability of the NPs to efficiently deliver the siRNA was validated in YTS‐2DL1 and primary human KIR2DL1^+^ NK (referred to as pNK KIR2DL1^+^). Indeed, treatment with NPs caused a significant reduction in the expression of SHP‐1 and Cbls in both YTS‐2DL1 and pNK KIR2DL1^+^ cells (Fig 4A).

We next examined the effect of NPs on NK cell effector function when stimulated with target cells. First, we determined the ability of NPs encapsulating SHP‐1 and Cbls siRNA to increase the intracellular calcium flux of YTS‐2DL1 cells incubated with 221‐Cw4 target cells. As shown in Fig 4B, calcium levels were markedly higher in YTS‐2DL1 pretreated with SHP‐1 and Cbls siRNA‐loaded NPs (red curve), relative to the levels observed in YTS‐2DL1 pretreated with N.S siRNA‐loaded NPs (blue curve). In addition, we incubated YTS‐2DL1 pretreated with SHP‐1 and Cbls or N.S siRNA‐loaded NPs with 221‐Cw4 target cells (Fig EV2C) and determined their ability to enhance NK cell degranulation and granzyme B release. YTS‐2DL1 pretreated with SHP‐1 and Cbls siRNA‐loaded NPs exhibited a significant fold increase of 1.728 ± 0.14 in their ability to secrete cytolytic granules following inhibitory interactions (721‐Cw4), compared to YTS‐2DL1 pretreated with N.S siRNA‐loaded NPs (*P* = 0.004; Fig 4C). Of note, treatment of YTS‐2DL1 with SHP‐1 and Cbls siRNA‐loaded NPs did not cause a significant increase in their degranulation following activating interactions (721‐Cw7), indicating the effect of these NPs is specific under inhibitory conditions (N.S siRNA‐loaded NPs/Cw7 1.53 ±0.14; SHP‐1 and Cbls siRNA‐loaded NPs/Cw7 1.86 ± 0.2, *P* = 0.38; Fig 4C). Additionally, YTS‐2DL1 cells pretreated with SHP‐1 and Cbls siRNA‐loaded NPs that were incubated with 221‐Cw4 cells exhibited a significant increase in the levels of secreted granzyme B compared to cells treated with NPs loaded with N.S siRNA (SHP‐1 and Cbls siRNA‐loaded NPs/Cw4, 186.83 ± 24.8 pg/ml; N.S siRNA‐loaded NPs/Cw4 118.95 ± 29.26 pg/ml, *P* = 0.0002; Fig 4D). The levels of CD107a and granzyme B secretion by YTS‐2DL1 cells pretreated with NPs loaded with SHP‐1 and Cbls siRNA incubated with 221‐Cw4 targets were similar to those incubated with 221‐Cw7 target cells (Fig 4C and D; CD107a: SHP‐1 and Cbls siRNA‐loaded NPs/Cw4 1.72 ± 0.14; N.S siRNA‐loaded NPs/Cw7, 1.53 ± 0.14, *P* = 0.66. Granzyme B: SHP‐1 and Cbls siRNA‐loaded NPs/Cw4 186.83 ± 24.8 pg/ml; N.S siRNA‐loaded NPs/Cw7 200.6 ± 33pg/ml, *P* = 0.4), indicating that downregulation of SHP‐1 and Cbls eliminates inhibitory signaling in these cells.

Finally, NK cell‐mediated killing of target cells was measured through a standard [^35^S] Met release assay. YTS‐2DL1 cells that were gene‐silenced for SHP‐1 and Cbls and incubated with 221‐Cw4 cells demonstrated a significant increase in cytotoxicity (SHP‐1 and Cbls siRNA‐loaded NPs/Cw4 31.89 ± 4.95%; N.S siRNA‐loaded NPs/Cw4 10.19 ± 0.7%, *P* = 0.03). YTS‐2DL1 cells that were pretreated with SHP‐1 and Cbls siRNA‐loaded NPs and subjected to inhibitory conditions demonstrated similar cytotoxic responses to cells treated with N.S siRNA under activating conditions (Fig 4E). Thus, these data demonstrate that gene silencing of inhibitory checkpoints SHP‐1 and Cbls in NK cells through NP administration results in a substantial increase of NK cell activation.

These data were verified in Primary NK isolated from peripheral blood mononuclear cells (PBMCs) following sort of KIR2DL1^+^ subpopulation. pNK KIR2DL1^+^ cells pretreated with SHP‐1 and Cbls siRNA‐loaded NPs exhibited a significant 2.06 ± 0.33 fold increase in their ability to secrete cytolytic granules following inhibitory interactions (221‐Cw4; Fig EV2D), in comparison with pNK cells pretreated with N.S siRNA‐loaded NPs (*P* = 0.016; Fig 4F). Interestingly, unlike YTS‐2DL1, pNK KIR2DL1^+^ cells pretreated with SHP‐1 and Cbls siRNA‐loaded NPs exhibited an increase in their ability to secrete cytolytic granules following activating interaction conditions as well, although the increase was not significant (N.S siRNA‐loaded NPs/Cw7 1.53 ± 0.11; SHP‐1 and Cbls siRNA‐loaded NPs/Cw7 2.65 ± 0.43, *P* = 0.083; Fig 4F). The differential receptor repertoire on pNKs is higher than on YTS‐2DL1, possibly explaining the differences between them.

### Lack of aberrant NK activation by NPs *in vitro*

**Figure 5:**
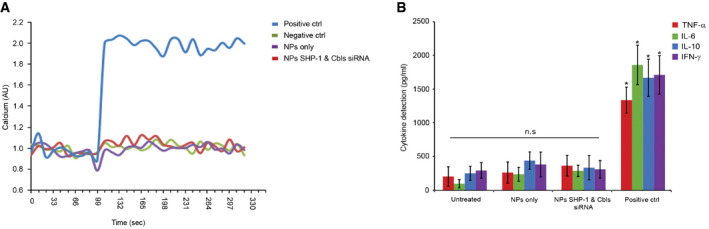
NKp46 antibody‐coated NPs do not induce spontaneous non‐specific NK cell activation or cytokine secretion YTS KIR2DL1 cells were incubated with SHP‐1 and Cbls siRNA‐loaded NPs (red curve) or empty NPs (purple curve). After 48 h, cells were loaded with Fluo‐3‐AM and analyzed for intracellular calcium levels for 20 min at 37°C. YTS KIR2DL1 cells that were treated with PBS served as a negative control (green curve). NK cells that were mixed with 721.221 Cw7 target cells served as a positive control (blue curve). Calcium levels were analyzed by spectrofluorometry (*n* = 3).Freshly isolated PBMCs were treated with SHP‐1 and Cbls siRNA‐loaded NPs, empty NPs, PBS as negative control, or PHA as a positive control. Following incubation, supernatant from each sample was collected and the levels of human cytokines TNF‐α, IL‐6, IL‐10, and IFN‐γ were determined using ELISA. The data represent four independent experiments (*n* = 4). *P* values were calculated by one‐way ANOVA following Tukey's *post hoc* analysis and are indicated by asterisks. *P ≤ 0.008. Data are shown as mean ± SEM. Data information: Exact *P* values are shown in Appendix Table S1. Source data are available online for this figure.

We next tested whether NKp46‐coated NPs are biologically inert and to rule out their potential to cause aberrant NK cell activation by measuring intracellular calcium flux in resting YTS‐2DL1 cells (Fig 5A). YTS cells incubated with 221‐Cw7 targets (positive control) showed higher calcium flux relative to the levels measured in naive YTS‐2DL1 (negative control), or in YTS‐2DL1 cells that were pretreated with empty NPs (NPs only) or with siRNA‐loaded NPs. These data indicate that NKp46 antibody‐coated NPs (either empty or loaded with siRNA) do not induce spontaneous intracellular calcium release in resting NK cells that are not activated against susceptible targets.

Next, we measured *in vitro* secretion of inflammatory cytokines in response to NP treatment. For these experiments, PBMCs were incubated with empty NPs, SHP‐1, and Cbls siRNA‐loaded NPs, PBS without NPs (untreated), or PHA and LPS as positive controls. The levels of human cytokines TNF‐α, IL‐6, IL‐10, and IFN‐γ were measured. As shown in Fig 5B, the NPs by themselves, either with or without siRNAs, did not induce significant differences in released cytokine concentrations relative to untreated cells, i.e., cells suspended in PBS without NPs, supporting the lack of aberrant activating activity of the NKp46 antibody‐coated NPs.

### The potential safety of systemic NP administration *in vivo*

**Figure EV3:**
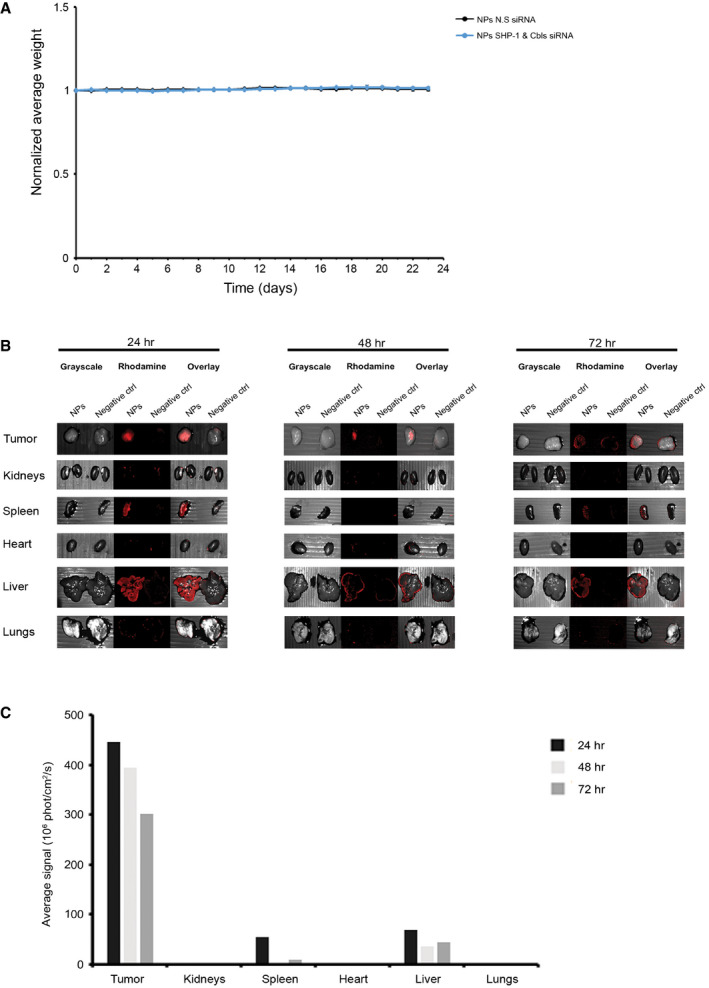
*In vivo* toxicity and biodistribution of NKp46 antibody‐coated NPs in tumor‐bearing NRG mice following efficacy study end point Diabetic/severe combined immunodeficiency (SCID/NOD) mice received intravenous (I.V) injection of NKp46 NPs every 72 h for a total of six injections. Mice were monitored for weight loss daily over the course of 23 days following the first treatment. Mice were treated with NK cells' gene silenced for SHP‐1 and Cbls using siRNA‐loaded NPs (*n* = 13) or N.S siRNA‐loaded NPs (*n* = 13) for three independent experiments.Mice were I.V administered fluorescently labeld NKp46‐NPs, or PBS as negative control. Mice were euthanized at indicated times following I.V injection of the NKp46‐NPs. Internal organs and tumors were taken out and imaged using computed tomography, CRi Maestro II.Calculation of average rhodamine fluorescence signal (10^6^ photon/cm^2^/s) per organ 24, 48, and 72 h from injection was performed using the Maestro software.

To determine the suitability of the NKp46 antibody‐coated NPs for *in vivo* studies, we examined whether these NPs induced stress‐related side effects in mice. Diabetic/severe combined immunodeficiency (SCID/NOD) mice were subcutaneously injected with 221‐Cw4 cells. When the tumor reached a volume of 70 mm^3^, the mice were randomly divided into two groups and received a total of six treatments by intravenous (I.V) injection of either SHP‐1 and Cbls siRNA‐loaded NPs, or N.S siRNA‐loaded NPs as a control group. There was no evidence of weight loss (Fig EV3A), lethargy, or other physical indicators of morbidity or stress over the course of 23 days following the first treatment in either of the groups.

### Molecularly modified NK cells gene silenced for SHP‐1 and Cbls via NKp46 antibody‐coated NPs suppress tumor growth *in vivo*

**Figure 6:**
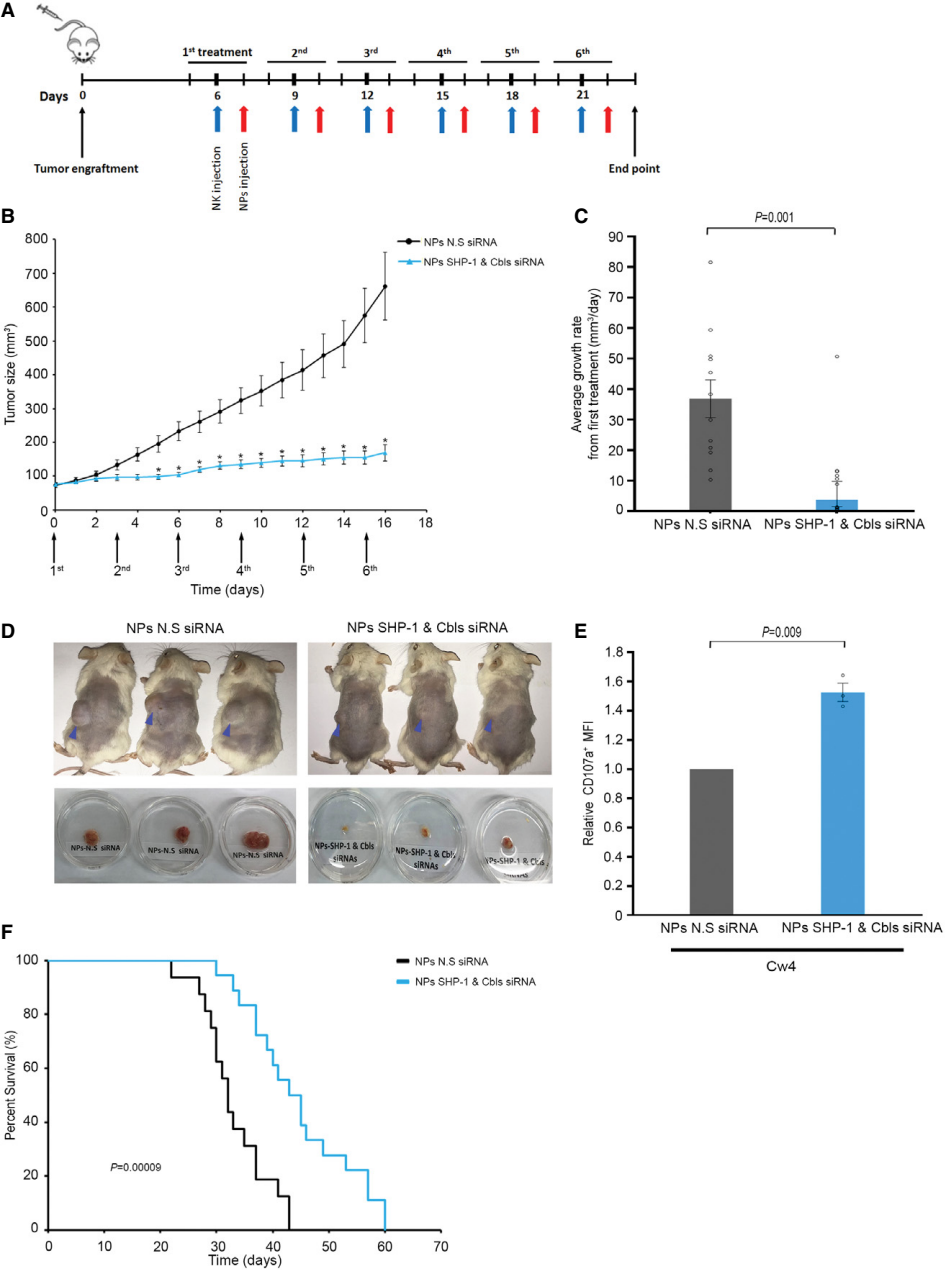
NK cells gene silenced for SHP‐1 and Cbls siRNA via NKp46 antibody‐coated NPs exhibit therapeutic efficacy in human B‐cell lymphoma engrafted mice Schematic representation of the experimental timeline.Tumor volumes measured daily in SCID/NOD mice treated with 3 × 10^6^ irradiated YTS KIR2DL1 and NPs containing Cbl‐b, c‐Cbl, and SHP‐1 siRNA (*n* = 13), or mice treated with 3 × 10^6^ irradiated YTS KIR2DL1, and NPs containing N.S siRNA that served as a negative control (*n* = 12). *P* values were calculated using two‐tailed Student's *t*‐test and are indicated by asterisks. *P < 0.05. Data are shown as mean ± SEM.Average growth rates of tumors from the first treatment to the end point from same experimental groups of mice as indicated in B. *P* value was calculated by two‐tailed Student's *t*‐test and is indicated within the graph *P* = 0.001. Data are shown as mean ± SEM.Images of the tumor‐bearing mice at the end point of the experiment (upper panel) and excised tumors (lower panels).Following 24 h after the last treatment, extracted cells from tumors of either SHP‐1 and Cbls or N.S siRNA‐loaded NKp46‐NPs‐treated mice were analyzed by flow cytometry. Expression of CD107a was measured by mean fluorescence intensity (MFI). Relative expression of CD107a MFI was normalized to the sample treated with N.S siRNA‐loaded NPs. The data represent three independent experiments (*n* = 3). Data are shown as mean ± SEM; *P* value was calculated by one‐sample *t*‐test and is indicated within the graph *P* = 0.009.Survival analysis in mice treated with NK cells' gene silenced for SHP‐1 and Cbls using siRNA‐loaded NPs (*n* = 18) or N.S siRNA‐loaded NPs (*n* = 18) as demonstrated by Kaplan–Meier survival curve. *P* values were calculated using the log‐rank test and is indicated within the graph *P* = 0.00009. Data information: Exact *P* values are shown in Appendix Table S1. Source data are available online for this figure.

Finally, we evaluated the efficacy of NP treatment in *in vivo* models. Human NK cells were injected into 221‐Cw4 engrafted mice followed by systemic delivery of siRNA‐loaded NPs as indicated in Fig 6A for a total of six treatments. Tumor volumes were monitored daily to examine the effect on tumor size and average growth rate (Fig 6B and C). Strikingly, mice treated with SHP‐1 and Cbls siRNA‐loaded NPs showed significantly smaller tumor volumes and reduced tumor growth rate compared to the control mice that were treated with N.S siRNA (Fig 6B–D).

To verify the specificity of NKp46 antibody‐coated NPs, we quantified the fluorescence signal of the NPs in the tumors and organs. Kidneys, spleen, heart, liver, and lungs were extracted 24–72 h following the last treatment. As shown in Fig EV3B and C, fluorescent NPs were predominantly found in the tumors, and hematopoietic tissues such as spleen and liver, with a very low presence in non‐hematopoietic tissues such as the kidneys and heart (Fig EV3C). These results support the efficient arrival and accumulation of the NPs in the tumor following injection of NKp46 antibody‐coated NPs to the bloodstream.

To characterize and elucidate the role of NK cells in tumor clearance, tumors were extracted 24 h following the final treatment and dissociated. Cells were then analyzed *ex vivo* by flow cytometry for their degranulation and the presence of rhodamine red labeled NPs. Strikingly, NK cells positive for SHP‐1 and Cbls siRNA‐loaded NPs showed significantly higher fold degranulation (1.52 ± 0.06, *P* = 0.009) compared to the N.S siRNA treated cells (Fig 6E).

**Figure EV4:**
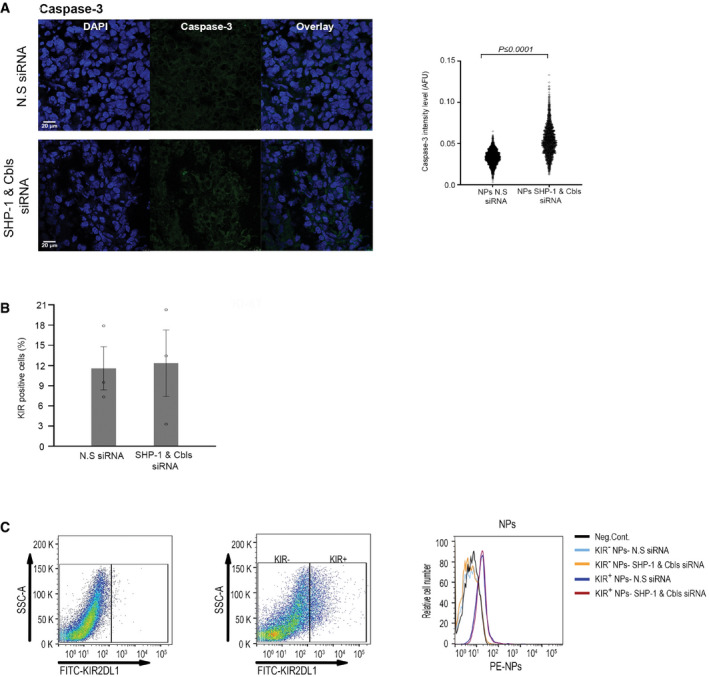
Anti‐tumor activity and activation of tumor‐infiltrating NK cells IHC analysis of apoptotic tumor cells via cleaved Caspase‐3 staining of tumor sections from NS (*n* = 1,875 cells) vs SHP‐1 and Cbls NPs (*n* = 1,477 cells) treated mice. *P* value was calculated by two‐tailed Student's *t*‐test and indicated within the graph *P* ≤ 0.0001.KIR expression on dissociated tumors to detect NK cells. Data are means ± SEM of three independent experiments (*n* = 3).FACS analysis of disassociated tumors from NS vs SHP‐1 and Cbls NPs treated mice. NK cells were identified and gated from single‐cell suspensions by staining for KIR2DL1, and analyzed for the percentage of NK cells incorporating fluorescently labeled NPs. Data information: Exact *P* values are shown in Appendix Table S1. Source data are available online for this figure

To further elucidate the impact of Cbls and SHP‐1 gene silencing in NK cells on the tumor cells, we examined the expression levels of cleaved Caspase‐3 as a marker of apoptosis using immunohistochemistry (IHC) in mice tumor sections. The tumor sections were stained with an antibody that recognizes the active cleaved form of Caspase‐3. A significant increase in apoptotic cells in sections from mice‐bearing tumors treated with NKp46 antibody‐coated NPs encapsulating SHP‐1 and Cbls siRNA (*P* < 0.0001, Fig EV4A) was measured relative to control xenografts. To evaluate the anti‐tumor activity of NK cells, cells extracted from tumors of NP‐treated mice were stained with anti‐KIR2DL1 antibodies to identify the percentage of NK cells in the tumor (Fig EV4B). FACS analysis demonstrated that most of the NK cells obtained from the tumors contained integrated NPs, as indicated by the rhodamine‐fluorescence labeling (Fig EV4C).

Finally, we studied the effects of NPs on the survival of engrafted mice. As demonstrated by the Kaplan–Meir curve (Fig 6F), NK cells gene‐silenced for SHP‐1 and Cbls significantly prolonged the survival of the mice compared to mice treated with NPs encapsulating NS siRNA (*P* = 0.00009).

In summary, our data demonstrate that lipid‐based NP targeting of NK cells greatly increased the cytotoxic potential of NK cells both *in vitro* and *in vivo*. Utilizing NPs to enhance the cytotoxic capacity of NK cells by downregulation of key intrinsic inhibitory checkpoint molecules may provide a robust immunotherapeutic approach.

## Discussion

Harnessing the power of the immune response to combat cancer has shown great benefit in the clinic, yet it is clear that current immunotherapeutic approaches have only limited efficacy against many types of cancer. Conventional immunotherapeutic treatments and specifically those directed against advanced/solid tumors have yet to demonstrate far‐reaching results on most patients without causing severe side effects (Martins *et al*, 2019). Thus, the challenge in the near future for immunotherapy is to develop cell‐specific strategies that effectively and safely augment current anti‐tumor responses.

NK cells are attractive immunotherapeutic candidates due to their innate ability to target tumors with their large receptor repertoire without the requirement for prior antigen exposure or antigen specificity (Di Vito *et al*, 2019). They can complement T‐cell immune surveillance by targeting MHC‐I‐deficient tumors, and recent data also demonstrate their ability to trigger substantial adaptive immune responses through cross‐talk with dendritic and T cells (Kalinski *et al*, 2005). Therefore, unleashing NK cell activity *in situ* may lead to robust and sustained activation of different immune cells in the TME, leading to more substantial clinical results in conjugation with current immunotherapeutic treatments.

Current strategies for harnessing NK cells, such as adoptive therapy and checkpoint blockade, have numerous disadvantages. These include the requirement for large‐scale NK cell‐expansion protocols for adoptive transfer with low efficiency, high risk of contamination, and potential oncogenic transformation due to viral transduction protocols. Adoptive transfer of genetically engineered cells through viral vector transduction is complex and has high production costs. The disadvantage of viral vectors is their random integration in the genome, causing to potential genetic abnormalities. Thus, it is essential to develop non‐viral vectors. Furthermore, systemic ICI treatment is accompanied by the risk of autoimmunity, in addition to adaptive and acquired resistance mechanisms, and the suppressive TME, which counteract the effects of treatment (Sutlu *et al*, 2009; Guillerey *et al*, 2016). Thus, to date, no clinical data have demonstrated significant benefits of harnessing NK cells in patients with solid tumors using available methods (Boyiadzis *et al*, 2017; Chidambaram *et al*, 2017). Nevertheless, it is possible that our novel approach to activate NK cells can be implemented in patients *in vivo*, to provide clinical benefits yet avoiding significant autoimmune side effects.

In this study, we targeted intracellular inhibitory signals that limit NK cell function against tumor cells *in vivo* without the need for *ex vivo* NK cell isolation. This approach takes advantage of the mechanisms of NK cell activation and inhibition together with our knowledge of the molecular processes, downstream to receptor engagement, which tune the NK cell activation threshold. Following the engagement of NK cell inhibitory receptors, cancer cell killing is suppressed by two main mechanisms: (i) dephosphorylation of signaling molecules by the protein tyrosine phosphatase (PTP), SHP‐1, and (ii) ubiquitylation‐mediated degradation of signaling molecules by the E3 ubiquitin ligases, Cbl‐b and c‐Cbl (Matalon *et al*, 2016). Abrogating expression of SHP‐1 and Cbls significantly reduces the NK cell activation threshold against cancerous cells, converting the inhibitory NK cell response into an activating one.

Here, we developed a gene silencing‐based approach, through delivery of lipid nanoparticle encapsulated siRNAs to target and gene‐silenced SHP‐1 and Cbls, which down modulate NK cell activation downstream to inhibitory receptor co‐ligation. This approach has advantages relative to other strategies by bypassing the large repertoire of inhibitory receptors expressed on NK cells, since SHP‐1 and Cbls inhibit NK cell activation downstream to multiple inhibitory checkpoint receptors such as PD‐1 (Chemnitz *et al*, 2004), TIGIT (Dougall *et al*, 2017), BTLA, CEACAM, TIM‐3, LAG‐3, and others (Watanabe *et al*, 2003; Torphy *et al*, 2017). Targeting inhibitory intracellular signaling moieties therefore eliminates the redundant activity of numerous receptors expressed on the NK cell surface (Angelo *et al*, 2015). Although we focus on a common immune‐editing mechanism that constrains NK cell activity, surface inhibitory receptors converge on common intracellular inhibitory signaling pathways, which may be relevant for various immune‐editing events. Thus, inhibition of the dominant negative intracellular signaling pathways may offer new opportunities to bypass the mechanisms of acquired resistance to ICI therapy.

Due to their small size, organic components, and negligible charge, NPs present a half‐life of 20–117 h in the blood circulation depending on the dose (Stathopoulos *et al*, 2005; Daraee *et al*, 2016) and they can penetrate passively into targeted NK cells. Thus, the NPs mainly reach NK cells distributed in peripheral blood, lymphoid tissues (e.g., lymph nodes, spleen), and non‐lymphoid tissues (e.g., lung and liver). Since NKp46 is upregulated on activated NK cells (Gauthier *et al*, 2019), and is stably expressed in the TME (Gauthier *et al*, 2019), NPs targeting this NK marker may diminish side effects and prevent damage to vital organs due to toxic by‐product accumulation of NPs in other tissues (Torchilin 2005; Daka *et al*, 2012). Moreover, the NKp46‐coated NPs are pre‐coated with hyaluronan, which as was previously shown by Bachar *et al* (2011) to have important roles including (i) serving as an efficient anchor for the antibody, and (ii) improving the blood circulation half‐life of the NP by providing a stealth coating layer onto the surface of the NP.

In general, the liposomal NPs used here possess excellent biodegradability and biocompatibility, making them an ideal carrier for siRNAs (Kulkarni *et al*, 2019). The use of siRNA for cancer therapeutics is increasing, and siRNA‐based therapy is starting to enter the clinical phase (Bumcrot *et al*, 2006; Iorns *et al*, 2007). Several siRNA‐based anti‐cancer drugs are currently being tested in clinical trials, and many are being actively sought for preclinical research (Collins *et al*, 2006; DeVincenzo *et al*, 2008; Resnier *et al*, 2013). These include novel targets that in many cases cannot be attacked with conventional drugs (Farag *et al*, 2002). Typically, these NPs are coated with the targeting ligand and loaded with an active compound, which provides more flexibility when compared to reagents such as Ontak^®^ and I^131^‐tositumomab in which the targeting and active agents must be covalently linked (Peer *et al*, 2003, 2008).

Here, we demonstrate the high targeting ability of lipid NPs by coating them with NKp46 antibody, increasing their delivery into NK cells in the TME. It is important to note that this system serves as a prototype which can be fine‐tuned to target specific subpopulations or additional markers to increase incorporation in to activated lymphocytes in the TME while avoiding naïve subsets and innate lymphoid cells, which also express NKp46. The NPs can be potentially coated with other antibodies, such as NKp44 ensuring exclusive targeting of activated T/NK cell subsets or other innate lymphoid populations. Functionally, the NKp46‐coated NPs demonstrated a striking ability to downregulate the expression of SHP‐1 and Cbls in NK cells, and consequently to significantly lower the activation threshold of NK cells and ensure an increase in NK cell cytotoxicity against inhibitory 221‐Cw4 targets (Fig 4). Critically, the NKp46‐coated NPs do not evoke non‐specific NK cell activation, nor induce dysregulated cytokine secretion (Fig 5). Furthermore, the efficacy of these NPs in activating NK cells *in vivo* was demonstrated. NK cells infiltrated by NKp46‐coated NPs greatly reduced tumor growth rate, and increased survival of tumor‐bearing mice (Fig 6). However, as the NK cells were injected I.T, it would be of a great interest to examine the efficacy other types of injections, such as I.V or I.P, considering the difficulty of NK cells to infiltrate solid tumors (Ben‐Shmuel *et al*, 2020).

The major limitation of most existing cancer therapy approaches is off‐target toxicity to healthy cells. Furthermore, most chemotherapy medications used in conservative therapeutic strategies are hydrophobic with low solubility, and high metabolic rate, thus, decreasing their biological availability, and leading to systemic toxicity (Lowenthal *et al*, 1996). The development of nanomedicine facilitates specific targeted delivery while minimizing the side effects of the anti‐cancer drugs and overcoming TME barriers. Several Food and Drug Administration (FDA) approved nanomedicines exist for selective cargo delivery, and recently, the first siRNA containing NP platform, Onpattro (patisiran), was granted FDA approval (Wood *et al*, 2018). Thus, NP‐mediated delivery may be more efficient than the currently used therapeutic approaches including chemotherapy or biological agents (such as antibodies) due to their potential to provide direct delivery of the therapeutic agents with significantly reduced side effects (Zhang *et al*, 2008).

The approach we describe here can be directed to a wide variety of immune targets and additional types of immune cells, enhancing anti‐cancer responses against a broad variety of malignancies. An important advantage of the NP system is its high versatility; the NPs can be loaded with different cargos, for example, small molecules, peptides, or proteins inhibitors, and can target numerous intracellular inhibitory molecules, such as SHP‐2, SHIP, Crk, and others (Galandrini *et al*, 2002; Peterson *et al*, 2008; Purdy *et al*, 2009). In addition, NPs can potentially be loaded with cargo to down modulate the NK immune response in patients with autoimmune and inflammatory pathologies (Zitti & Bryceson, 2018), as well as additional immune cell subsets.

The field of immunotherapy is moving in the direction of combinatorial therapies, including both immunotherapeutic approaches and conventional treatments (Joshi *et al*, 2019). Our novel strategy can possibly augment combinatorial therapies and enhance anti‐cancer drug delivery. In future, NPs can also be constructed in a tailored fashion, suiting the specific NK profile of a given patient (Paucek *et al*, 2019). This precision approach can greatly increase the efficacy of treatment while reducing unwanted side effects due to particular expression patterns of immune receptors in different patients.

## Materials and Methods

### Cells

The cell lines used include the human YTS NK cell line, expressing the inhibitory receptor KIR2DL1 (YTS KIR2DL1); two variants of the human 721.221 B‐lymphoblastoid cell line, 721.221‐HLA‐Cw4, and 721.221‐HLA‐Cw7. These cells were kindly provided by Prof. Ofer Mandelboim (Department of Microbiology and Immunology, Faculty of Medicine, Hebrew University of Jerusalem, Israel). YTS KIR2DL1 cells were cultured in Iscove's medium supplemented with 10% fetal bovine serum (FBS), 2 mM l‐glutamine, 50 μg/ml penicillin, 50 μg/ml streptomycin and 50 μM 2‐mercapto‐ethanol; 721.221‐HLA‐Cw4, and 721.221‐HLA‐Cw7 expressing mCherry fluorescent protein; human erythroleukemia, K562. 721.221‐HLA‐Cw4/7 and K562 cells were cultured in RPMI supplemented with 10% FBS, 2 mM l‐glutamine, 50 μg/ml penicillin, 50 μg/ml streptomycin, 1% non‐essential amino acids, and 1% sodium pyruvate. NK92, a human NK lymphoma cell line; NK92 cells expressing recombinant NKp46 were kindly provided by Prof. Angel Porgador (Department of Microbiology, Immunology and Genetics, Faculty of Health Sciences, Ben‐Gurion University of the Negev, Beer Sheva, Israel), and NK92 cell lines were cultured in Alpha‐MEM medium supplemented with 10% horse serum, 10% FBS, 0.2 mM myo‐inositol, 0.1 mM β‐mercaptoethanol, 0.02 mM folic acid, 1% penicillin/streptomycin, and 100 IU/ml of recombinant human IL‐2. All the cell lines were tested negative for mycoplasma contamination.

### PBMCs isolation

Human primary PBMCs were isolated from whole blood of healthy donors, as previously described (Barda‐Saad *et al*, 2005). Blood samples from healthy donors were randomly collected and provided by Magen David Adom (MDA; Israeli National Blood Bank). Informed consent was obtained from all donors. Donor's identification information remained anonymous. The experiments conformed to the principles set out in the WMA Declaration of Helsinki and the Department of Health and Human Services Belmont Report. The research was performed with approval of and according to the guidelines of the Bar‐Ilan University Ethics committee. Briefly, human PBMCs were isolated from whole blood by Ficoll‐Histopaque density gradient centrifugation (MP Biomedical). Isolated cells were cultured in RPMI 1640 (Sigma‐Aldrich) supplemented with 10% FBS (Biological industries). All cells were grown at 37°C, under an atmosphere containing 5% CO_2_.

### Primary NK KIR2DL1^+^ cell isolation

Primary NK KIR2DL1^+^ cells were isolated as previously described (Matalon *et al*, 2018). Briefly, primary NK cells were isolated from PBMCS of healthy donors using the EasySep™ human NK cell enrichment kit (STEMCELL Technologies). Subsequently, KIR2DL1‐expressing cells were isolated by staining the entire NK cell population with anti‐KIR2DL1‐PE antibody (Miltenyi Biotec), followed by magnetic separation using the EasySep™ human PE selection kit (STEMCELL Technologies) according to the manufacturer's instructions. NK cell isolation efficiencies were >95%.

### Antibodies

Rabbit anti‐SHP‐1 (C‐19), rabbit anti‐GAPDH (FL‐335; Santa Cruz Biotechnology), mouse anti‐Cbl‐b (Santa Cruz Biotechnology), rabbit anti‐Cbl (Santa Cruz Biotechnology), mouse anti‐CD107a (Bio Legend), mouse anti‐CD107a‐FITC (Bio Legend), mouse anti‐KIR2DL1 (Bio Legend), Alexa Fluor 647‐conjugated goat anti‐rabbit (Invitrogen). Antibodies details are shown in Appendix Table S2

### RNA interference

siRNAs specific for human c‐Cbl or Cbl‐b, SHP‐1, and non‐specific control siRNA were purchased from Sigma‐Aldrich. For knockdown of Cbl proteins, specific oligonucleotides encoding siRNAs were as follows: c‐Cbl, sense 5′‐CUGUUGACAGACAGACUAA‐3′, anti‐sense 5′‐UUAGUCUGUCUGUCAACAG‐3′; Cbl‐b, sense 5′‐CCCUUAUUUCAAGCCCUGA‐3′, anti‐sense 5′‐UCAGGGCUUGAAAUAAGGG‐3′. For knockdown of SHP‐1, the siRNA were as follows: sense, 5′‐CCCUGACCCUGUGGAAGCA, anti‐sense 5′‐UGCUUCCACAGGGUCAGGG‐3′, Non‐targeting (non‐specific), negative control siRNA duplexes were as follows: sense 5′‐UAGCGACUAAACACAUCAA‐3′, anti‐sense 5′‐UUGAUGUGUUUAGUCGCUA‐3′.

### Transfections by electroporation (AMAXA)

Transfections of Cbl‐b, c‐Cbl, SHP‐1 and non‐specific siRNAs were conducted by using a Nucleofector^®^ Kit & device from AMAXA. Transfections were conducted 48 h before biochemical and functional experiments.

### Lysis of NK cells

Cells were centrifuged and resuspended in 20 μl of lysis buffer. Cells were then placed on ice for 30 min and subsequently centrifuged at 14,000 rpm (or 20,000 *g*). The supernatant containing the cell lysate was collected, and sample buffer (5X) was added to the cell lysates. Samples were heated at 100°C, for 5 min, followed by centrifugation.

### Western blot

Western blot (WB) and stripping of membranes were performed as previously described (Matalon *et al*, 2018). Protein samples were resolved by sodium dodecyl sulfate‐polyacrylamide gel electrophoresis (SDS–PAGE), transferred to nitrocellulose membranes, and analyzed by Western blotting with the appropriate primary antibodies. Immunoreactive proteins were detected with horseradish peroxidase (HRP)‐coupled anti‐mouse or anti‐rabbit secondary antibody followed by detection with enhanced chemiluminescence (PerkinElmer). For WCLs, band intensities were measured by densitometric analysis with ImageJ software and were expressed relative to the intensities of bands corresponding to GAPDH, which was the loading control. For immunoprecipitated samples, band intensities were measured by densitometric analysis with ImageJ software and were expressed relative to those of the precipitation control.

### Ca^2+^ release assay

YTS cells (0.5 × 10^6^ to 1 × 10^6^) were incubated with 1 μg calcium‐sensitive dye, Fluo‐3‐AM per sample in RPMI 1640 medium at 37°C for 45 min. The cells were washed and resuspended in RPMI 1640 without phenol red containing 10 mM HEPES and 0.5 mM probenecid, and were maintained at room temperature for 20 min. The cells were incubated at 37°C for 5 min before measurements and first analyzed for 100 s to establish the basal intracellular Ca^2+^concentration, followed by mixing at a 1:1 ratio with target cells. Ca^2+^ influx was measured using a Synergy 4 spectrophotometer microplate reader (BioTek).

### CD107a assay

YTS cells (3 × 10^5^) were co‐incubated with 6 × 10^5^ 721.221 target cells at 37°C for 2 h in the presence of monensin (BioLegend). The cells were centrifuged, incubated with anti‐CD107a antibody for 30 min on ice, and washed twice. Staining with isotype‐specific, Alexa flour‐conjugated antibody was then performed on ice for 30 min. Cells were washed twice and analyzed by flow cytometry. As a negative control, cells were incubated with fluorescent secondary antibody alone.

Primary NK KIR2DL1^+^ cells (2 × 10^5^) were co‐incubated with 4 × 10^5^ 721.221 target cells at 37°C for 5 h in the presence of monensin (BioLegend). The cells were centrifuged and incubated with anti‐CD107a‐FITC‐conjugated antibody for 30 min on ice. The cells were washed twice and analyzed by flow cytometry.

### Cytotoxicity assay

The cytolytic activity of NK cells against target cells was determined using a standard [^35^S] Met release assay and was performed as previously described (Matalon *et al*, 2018).

### Granzyme B release assay

A granzyme B release assay was conducted using Human Granzyme ELISA development Kit (Mabtech). ELISA reading was performed at 450 nm using a synergy 4 spectrophotometer.

### Flow cytometric analysis for mAb NKp46 staining

3 × 10^5^ NK cells were incubated for 30 min on ice with 50 µl culture supernatant from anti‐NKp46 monoclonal antibody producing hybridomas. The cells were then washed twice with PBS and incubated for 30 min on ice with goat anti‐mouse IgG secondary antibody. The cells were washed twice and analyzed by flow cytometry.

### Nanoparticle preparation

Four lipids were mixed at 6:2:1.9:0.1 molar ratios: (i) Phosphatidylcholine (PC), (ii) cholesterol (Chol), (iii) 1, 2‐dihexadecanoyl‐sn‐glycero‐3‐phosphoethanolamine (DPPE), and (iv) DPPE labeled with rhodamine red (DPPE‐PE, excitation/emission: 560/583 nm; Avanti). The lipid mixture was frozen in liquid nitrogen and then heated to 37°C. Liposomes were coated with 6 mg hyaluronic acid (HA; high molecular weight, R&D) and 40 mg of 1‐ethyl‐3‐(3‐dimethylaminopropyl) carbodiimide (EDAC; Sigma). For each 50 µg of liposomes, 400 mM of EDAC and 100 mM of N‐Hydroxysuccinimide (NHS) were added (Sigma). The solution was incubated with 50 µg of NKp46 antibodies at room temperature overnight. The liposomes were then lyophilized overnight to yield a nanoparticle powder.

### Entrapment efficiency of siRNA

Non‐specific siRNA (N.S siRNA) labeled with FITC was mixed with full‐length recombinant protamine (Sigma‐Aldrich) at a 1:5 siRNA:protamine molar ratio, in nuclease free water, and incubated for 20 min at RT to form a complex. Immediately before use, the lyophilized NPs (300 μg total lipids) were rehydrated by adding 0.2 ml nuclease‐free water containing protamine‐condensed siRNA.

### Entrapment of siRNA

The amount of the NPs and siRNA that used was dependent on the cell number. Every 1 × 10^6^ cells were incubated with 150 µg NPs loaded with 750 pmol siRNA (250 pmol/each of SHP‐1, Cbl‐b and c‐Cbl siRNA),

### Cytokine release assay

Freshly isolated PBMCs from healthy donors (1 × 10^6^ cells/ml) were incubated with empty NPs; SHP‐1 and Cbls siRNA‐loaded NPs; PBS (untreated); or PHA and LPS as positive controls. Following incubation, the supernatant from each sample was collected. The levels of human cytokines TNF‐α, IL‐6, IL‐10, and IFN‐γ were determined using human Mini ELISA Development kits (PeproTech).

### Confocal microscopy

Cells were incubated with 50 µg rhodamine‐labeled NPs for 24 h. Cells were then centrifuged in a 24‐well plate onto pol‐l‐lysine‐treated cover slips for 7 min at 300 *g*. Following centrifugation, the cover slips containing the cells were washed, fixed with paraformaldehyde (3.7%), and counter stained with 50 ng DAPI (Molecular probes), and images were acquired using the Leica TCS SP8 confocal microscope under a 63×objective lens.

### Immunohistochemistry

Tumor tissues were harvested from mice and were cut into 5‐mm‐thick tissue blocks. The tissue blocks were covered with O.C.T. cryo‐embedding media and were frozen with 2‐methyl‐butane cooled by liquid nitrogen. The frozen tissue blocks were sliced into 8‐µm sections and mounted on glass slides (SuperFrost Plus, Thermo Scientific). Sections were fixed in cold acetone for 10 min, dried, incubated in blocking solution (10% FCS, 1% BSA dissolved in 0.01 M phosphate‐buffered saline [PBS], pH 7.2) for 30 min, and then incubated overnight at 4°C with rabbit anti‐cleaved Caspase‐3 (Cell Signaling) dissolved in PBS with 0.5% BSA. The tissue sections were rinsed twice in PBS, and once in PBS with 0.1% Tween, followed by incubation with secondary antibodies (Alexa Fluor 594‐conjugated goat anti‐rabbit) in PBS with 0.5% BSA for 45 min at R.T. Tissue sections were then rinsed twice in PBS and once in PBS with 0.1% Tween, followed by incubation with 0.3 µM DAPI (Invitrogen) in PBS for 15 min at R.T. Finally, the sections were rinsed twice in PBS and mounted with mounting medium. Images were acquired with the Leica SP8 confocal inverted microscope under a 63× objective lens.

### Mice

Male SCID/NOD mice, 6–8 weeks, were purchased from the Jackson Labs. All mice used were from colonies that were inbred and maintained under SPF conditions at the Bar‐Ilan animal house. Housing and breeding of mice and experimental procedures were performed according to the guidelines of the Bar‐Ilan University Ethics Committee. Mice were subcutaneously injected with 3 × 10^6^ 721.221 human B‐cell lymphoma (221.Cw4‐HLA cells) in PBS mixed with Matrigel (CORNING; 1:1 v/v ratio). Mouse weight, signs of stress or pain, and tumor volume were evaluated daily. Tumor volume was measured using a digital caliper, and the following formula was used to calculate the tumor volume: Volume (mm^3^) = 0.5 × (major axis) × (minor axis)^2^ (Tomayko *et al*, 1989). Tumor average growth rate was calculated according to the formula: Average growth rate = (Current tumor size − Initial tumor size)/Elapsed treatment time (days) (Tomayko *et al*, 1989).

### *In vivo* efficacy studies

After tumors in xenograft mice reached a volume of 70 mm^3^, mice were randomly divided into two groups and were treated intratumorally (I.T), with irradiated human YTS‐2DL1 NK cells, and intravenously (I.V) with 300 μg NPs loaded with 1,500 pmol siRNA (500 pmol/each of SHP‐1, Cbl‐b, and c‐Cbl), every 72 h for a total of six treatments. Tumor volumes were measured daily using a digital caliper. At 24 h following the last injection, mice were euthanized by CO_2_, and tumors were harvested.

### Statistical analysis

Data are expressed as mean±standard error of the mean (SEM). Statistically significant differences between the groups were determined as follows:

Independent sample *t*‐tests were used when comparing two independent groups. One‐way ANOVA was used if more than two groups were compared, followed by Tukey's multiple comparisons test. In cases of two factors, a two‐way ANOVA was used.

For data normalized to a reference group, a logarithmic transformation was applied prior to analysis, to obtain log‐fold changes. Then, groups were compared to the reference with one‐sample *t*‐tests (against a mean of 0) and were compared with each other with either an independent sample *t*‐test or one‐way ANOVA (depending on the number of groups). Finally, when multiple tests were performed, *P* values were corrected for multiple testing using the Benjamini–Hochberg (FDR) correction.

Mean differences were considered as statistically significant when *P* values were smaller than a significance level of 0.05.

Normality and homoscedasticity assumptions were checked graphically by inspecting residual plots obtained from ANOVA models (e.g., residual quantile‐quantile (QQ) plot, residual vs fitted values, etc.)

All analyses were conducted in the R statistical environment (R Core Team, 2021).

## Author contributions

MB‐S, GB, and BS designed the research. GB, BS, AR, AB‐S, AP, TJ, ML, and SK performed the experiments. GB, BS, AR, AB‐S, AP, and MB‐S analyzed the data. JBIC performed statistical analysis. MBS wrote the manuscript.

## Conflict of interest

The authors declare that they have no conflict of interest.

## Supporting information



Appendix

Expanded View Figures PDF

Source Data for Expanded View

Source Data for Figure 1

Source Data for Figure 4

Source Data for Figure 5

Source Data for Figure 6

## Data Availability

This study includes no data deposited in external repositories.
