# Supplementary material for: Modulation of intrinsic inhibitory checkpoints using nano‐carriers to unleash NK cell activity
Source: EMBO Mol Med. 2021 Nov 2;14(1):e14073. doi: 10.15252/emmm.202114073 (PMC8749471; doi:10.15252/emmm.202114073)
Supplement: Supplementary file 1 — Appendix [file EMMM-14-e14073-s008.pdf]

Appendix

Modulation of intrinsic inhibitory checkpoints using nanocarriers to unleash NK cell activity

Guy Biber[#], Batel Sabag[#], Anat Raiff, Aviad Ben-Shmuel, Abhishek Puthenveetil, Jennifer Benichou Israel Cohen, Tammir Jubany, Moria Levy, Shiran Killner, and Mira Barda-Saad[1]*

Affiliations:

[1]The Mina and Everard Goodman Faculty of Life Sciences, Bar-Ilan University, Ramat-Gan 5290002, Israel.

[#] These authors contributed equally to this manuscript.

[*]**Address correspondence to:**
Prof. Mira Barda-Saad

The Mina and Everard Goodman Faculty of Life Sciences

Bar-Ilan University

Ramat-Gan 5290002, Israel

Tel.: +972-3-5317311; Fax: +972-3-7384058; E-mail: Mira.Barda-Saad@biu.ac.il

Table of contents:

Appendix Table S1 **Page 2**

Appendix Table S2 **Page 7**

Appendix Table S1

Exact statistical test results for indicated figures.

Figure 1A					
**comparison**	**test**	**pval**	**padj**		
Ctrl - SHP-1	1-sample t-test	0.0156	0.0156		
Ctrl - Cbl-b	1-sample t-test	0.0097	0.0145		
Ctrl - c-Cbl	1-sample t-test	0.0076	0.0145		
Figure 1C					
**comparison**	**test**	**pval**	**padj**		
MOCK _CW4_MOCK _CW7	1-sample t-test	0.0005	0.0016		
MOCK _CW4_NS_CW4	1-sample t-test	0.4916	0.4916		
MOCK _CW4_SHP-1 and Cbls siRNA _CW4	1-sample t-test	0.027	0.0404		
ALL	1-way ANOVA	0.0099	NA		
MOCK _CW7-SHP-1 and Cbls siRNA _CW4	Tukey	NA	0.987		
NS_CW4-SHP-1 and Cbls siRNA _CW4	Tukey	NA	0.0177		
NS_CW4-SHP-1 and Cbls siRNA _CW7	Tukey	NA	0.0181		
Figure 1D					
**comparison**	**test**	**pval**	**padj**		
ALL	1-way ANOVA	0.0039	NA		
Mock _CW4-SHP-1 and Cbls siRNA _CW4	Tukey	NA	0.0222		
Mock _CW7-SHP-1 and Cbls siRNA _CW4	Tukey	NA	0.9321		
NS_CW4-SHP-1 and Cbls siRNA _CW4	Tukey	NA	0.0349		
Mock _CW7-Mock _CW4	Tukey	NA	0.0133		
NS_CW4-Mock _CW4	Tukey	NA	0.9503		
NS_CW4-Mock _CW7	Tukey	NA	0.02		
Figure 1E					
**comparison**	**test**	**pval**	**padj**		
ALL	1-way ANOVA	0.0002	NA		
Mock _CW4-SHP-1 and Cbls siRNA _CW4	Tukey	NA	0.0119		
Mock _CW7-SHP-1 and Cbls siRNA _CW4	Tukey	NA	0.9106		
N.S._CW4-SHP-1 and Cbls siRNA _CW4	Tukey	NA	0.0101		
Mock _CW7-Mock _CW4	Tukey	NA	0.0035		
N.S._CW4-Mock _CW4	Tukey	NA	0.9991		
N.S._CW4-Mock _CW7	Tukey	NA	0.003		
Figure 4A YTS					

comparison	test	pval	padj		
control-SHP-1	1-sample t-test	0.0024	0.0049		
control-Cbl-b	1-sample t-test	0.0032	0.0049		
control-c-Cbl	1-sample t-test	0.0104	0.0104		
Figure 4A pNK					
**comparison**	**test**	**pval**	**padj**		
Ctrl - SHP-1	1-sample t-test	0.0126	0.0126		
Ctrl - Cbl-b	1-sample t-test	0.0035	0.0053		
Ctrl - c-Cbl	1-sample t-test	0.0014	0.0042		
Figure 4C					
**comparison**	**test**	**pval**	**padj**		
control-SHP-1	1-sample t-test	0.0024	0.0049		
control-Cbl-b	1-sample t-test	0.0032	0.0049		
control-c-Cbl	1-sample t-test	0.0104	0.0104		
Figure 4D					
**comparison**	**test**	**pval**	**padj**		
ALL	1-way ANOVA	0.0001	NA		
NS_Cw4-Mix_Cw4	Tukey	NA	0.0022		
NS_Cw4-NS_Cw7	Tukey	NA	0.0031		
Mix_Cw4-NS_Cw7	Tukey	NA	0.9996		
Figure 4E					
**comparison**	**test**	**pval**	**padj**		
ALL	1-way ANOVA	0.0308	NA		
NS_CW4-SHP-1 and Cbls siRNA _CW4	Tukey	NA	0.0321		
NS_CW7-SHP-1 and Cbls siRNA _CW4	Tukey	NA	0.7625		
NS_CW7-NS_CW4	Tukey	NA	0.0775		
Figure 4F					
**comparison**	**test**	**pval**	**padj**		
NS_CW4_SHP-1 and Cbls siRNA _CW4	1-sample t-test	0.0169	0.0169		
NS_CW4_NS_CW7	1-sample t-test	0.0052	0.0102		
NS_CW4_SHP-1 and Cbls siRNA _CW7	1-sample t-test	0.0068	0.0102		
ALL	1-way ANOVA	0.0991	NA		
SHP-1 and Cbls siRNA _CW7-SHP-1 and Cbls siRNA _CW4	Tukey	NA	0.4977		
NS_CW7-SHP-1 and Cbls siRNA _CW4	Tukey	NA	0.4674		
NS_CW7-SHP-1 and Cbls siRNA _SHP-1 and Cbls siRNA	Tukey	NA	0.0831		
Figure 5B IL-10					
**comparison**	**test**	**pval**	**padj**		
ALL	1-way ANOVA	0.0004	NA		

NPs only-NPs-SHP-1 and Cbls siRNA	Tukey	NA	0.9772		
Positive ctrl (PHA)-NPs-SHP-1 and Cbls siRNA	Tukey	NA	0.0013		
Untreated-NPs-SHP-1 and Cbls siRNA	Tukey	NA	0.9877		
Positive ctrl (PHA)-NPs only	Tukey	NA	0.0025		
Untreated-NPs only	Tukey	NA	0.8853		
Untreated-Positive ctrl (PHA)	Tukey	NA	0.0008		
Figure 5B IFN- γ					
**comparison**	**test**	**pval**	**padj**		
ALL	1-way ANOVA	0.0001	NA		
NPs only-NPs-siRNA	Tukey	NA	0.9954		
Positive ctrl (PHA)-NPs-siRNA	Tukey	NA	0.0004		
Untreated-NPs-siRNA	Tukey	NA	1		
Positive ctrl (PHA)-NPs only	Tukey	NA	0.0007		
Untreated-NPs only	Tukey	NA	0.9919		
Untreated-Positive ctrl (PHA)	Tukey	NA	0.0003		
Figure 5B IL-6					
**comparison**	**test**	**pval**	**padj**		
ALL	1-way ANOVA	0	NA		
NPs only-NPs-SHP-1 and Cbls siRNA	Tukey	NA	0.9962		
Positive ctrl (PHA)-NPs-SHP-1 and Cbls siRNA	Tukey	NA	0		
Untreated-NPs-SHP-1 and Cbls siRNA	Tukey	NA	0.8422		
Positive ctrl (PHA)-NPs only	Tukey	NA	0		
Untreated-NPs only	Tukey	NA	0.9288		
Untreated-Positive ctrl (PHA)	Tukey	NA	0		
Figure 5B TNF-α					
**comparison**	**test**	**pval**	**padj**		
ALL	1-way ANOVA	0.0004	NA		
NPs only-NPs-SHP-1 and Cbls siRNA	Tukey	NA	0.9689		
Positive ctrl (PHA)-NPs-SHP-1 and Cbls siRNA	Tukey	NA	0.0032		
Untreated-NPs-SHP-1 and Cbls siRNA	Tukey	NA	0.8981		
Positive ctrl (PHA)-NPs only	Tukey	NA	0.0013		
Untreated-NPs only	Tukey	NA	0.9947		
Untreated-Positive ctrl (PHA)	Tukey	NA	0.0008		
Figure 6B					
0	First injection	independent samples t-test	0.7623		

1		independent samples t-test	0.6300		
2		independent samples t-test	0.3484		
3	Second injection	independent samples t-test	0.0517		
4		independent samples t-test	0.0068		
5		independent samples t-test	0.0024		
6	Third injection	independent samples t-test	0.0009		
7		independent samples t-test	0.0010		
8		independent samples t-test	0.0007		
9	Forth injection	independent samples t-test	0.0005		
10		independent samples t-test	0.0006		
11		independent samples t-test	0.0007		
12	Fifth injection	independent samples t-test	0.0008		
13		independent samples t-test	0.0006		
14		independent samples t-test	0.0005		
15	Sixth injection	independent samples t-test	0.0003		
16		independent samples t-test	0.0004		
Figure 6C					
**comparison**	**test**	**pval**			
NPs N.S siRNA - NPs SHP-1 Cbls<br><br>siRNAs	independent samples t-test	0.001			
Figure 6E					
**comparison**	**test**	**pval**			
N.S siRNA - SHP-1 and Cbls siRNA	1-sample t-test	0.0092			
Figure 6F					
Log rank test					
	N	Observed	Expected	(O-E)^2/E	(O-E)^2/V
condition=MIX	18	18	26.5	2.73	15.3
condition=N.S	16	16	7.5	9.62	15.3

Chisq= 15.3 on 1 degrees of freedom, p=0.00009					
Figure EV1A					
**comparison**	**test**	**pval**	**padj**		
Mock - 250pmol Cbl-b siRNA	1-sample t-test	0.0071	0.0212		
Mock - 500pmol Cbl-b siRNA	1-sample t-test	0.02	0.03		
250pmol Cbl-b siRNA - 500pmol Cbl-b siRNA	independent samples t-test	0.0513	0.0513		
Figure EV1B					
**comparison**	**test**	**pval**	**padj**		
Mock - 250pmol c-Cbl siRNA	1-sample t-test	0.0151	0.0226		
Mock - 500pmol c-Cbl siRNA	1-sample t-test	0.0052	0.0155		
250pmol c-Cbl siRNA - 500pmol c-Cbl siRNA	independent samples t-test	0.0314	0.0314		
Figure EV1C					
**comparison**	**test**	**pval**	**padj**		
Mock - 250pmol SHP-1 siRNA	1-sample t-test	0.0104	0.0256		
Mock - 500pmol SHP-1 siRNA	1-sample t-test	0.0171	0.0256		
250pmol SHP-1 siRNA - 500pmol SHP-1 siRNA	independent samples t-test	0.8872	0.8872		
Figure EV1D					
**comparison**	**test**	**pval**	**padj**		
YTS NS siRNA - YTS SHP-1 siRNA	1-sample t-test	0.0157	0.0391		
YTS NS siRNA - YTS Cbl-b siRNA	1-sample t-test	0.0356	0.0593		
YTS NS siRNA - YTS c-Cbl siRNA	1-sample t-test	0.0629	0.0629		
YTS NS siRNA - YTS Cbl-b+c-Cbl siRNA	1-sample t-test	0.0479	0.0598		
YTS NS siRNA - YTS SHP-1+Cbls siRNA	1-sample t-test	0.0015	0.0073		
Figure EV4A					
**comparison**	**test**	**pval**			
Caspase 3_NS - Caspase 3_CBL SHP1	independent samples t-test	0.00000			
Figure EV4B					
**comparison**	**test**	**pval**			
N.S - SHP-1 and Cbls siRNA	independent samples t-test	0.9037			

Appendix Table S2

Antibody details of all antibodies used in this research.

Antigen /marker	Fluorochrome	Species/ host	Clone	Dilution	Vendor	Catalog no	Application
SHP-1	-	Rabbit	C-19	1/1000	Santa Cruz	SC-287	WB
GAPDH	-	Rabbit	FL-335	1/1000	Santa Cruz	SC-25778	WB
Cbl- b	-	Mouse	G-1	1/250	Santa Cruz	SC-8006	WB
Cbl	-	Mouse	A-9	1/250	Santa Cruz	SC-1651	WB
CD107a	-	Mouse	H4A3	1/20	Bio Legend		FC
CD107a	FITC	Mouse	H4A3	1/20	Bio Legend	328606	FC
Alexa Fluor 647-conjugated Goat anti-Mouse	Alexa Fluor 647	Goat	-	1/2500	Invitrogen	A21235	FC
Alexa Fluor 594-conjugated Goat anti-Rabbit	Alexa Fluor 594	Goat	-	1/2000	Invitrogen		FC
KIR2DL1/S1	-	Mouse	HP-MA4	1/100	Bio Legend	339502	FC
KIR2DL1/S1	PE	Mouse		1/100	Miltenyi	130099209	FC
Caspase 3	-	Rabbit	5AIE	1/1000	Bio Legend	9664	IHC
CD56	FITC	Mouse	HCD56	1/50	Bio Legend	318303	FC
CD3	PE-Cy5	Mouse		1/100	BD	555334	FC
