# Supplementary material for: Modulation of intrinsic inhibitory checkpoints using nano‐carriers to unleash NK cell activity
Source: EMBO Mol Med. 2021 Nov 2;14(1):e14073. doi: 10.15252/emmm.202114073 (PMC8749471; doi:10.15252/emmm.202114073)
Supplement: Supplementary file 2 — Expanded View Figures PDF [file EMMM-14-e14073-s003.pdf]

Expanded View Figures

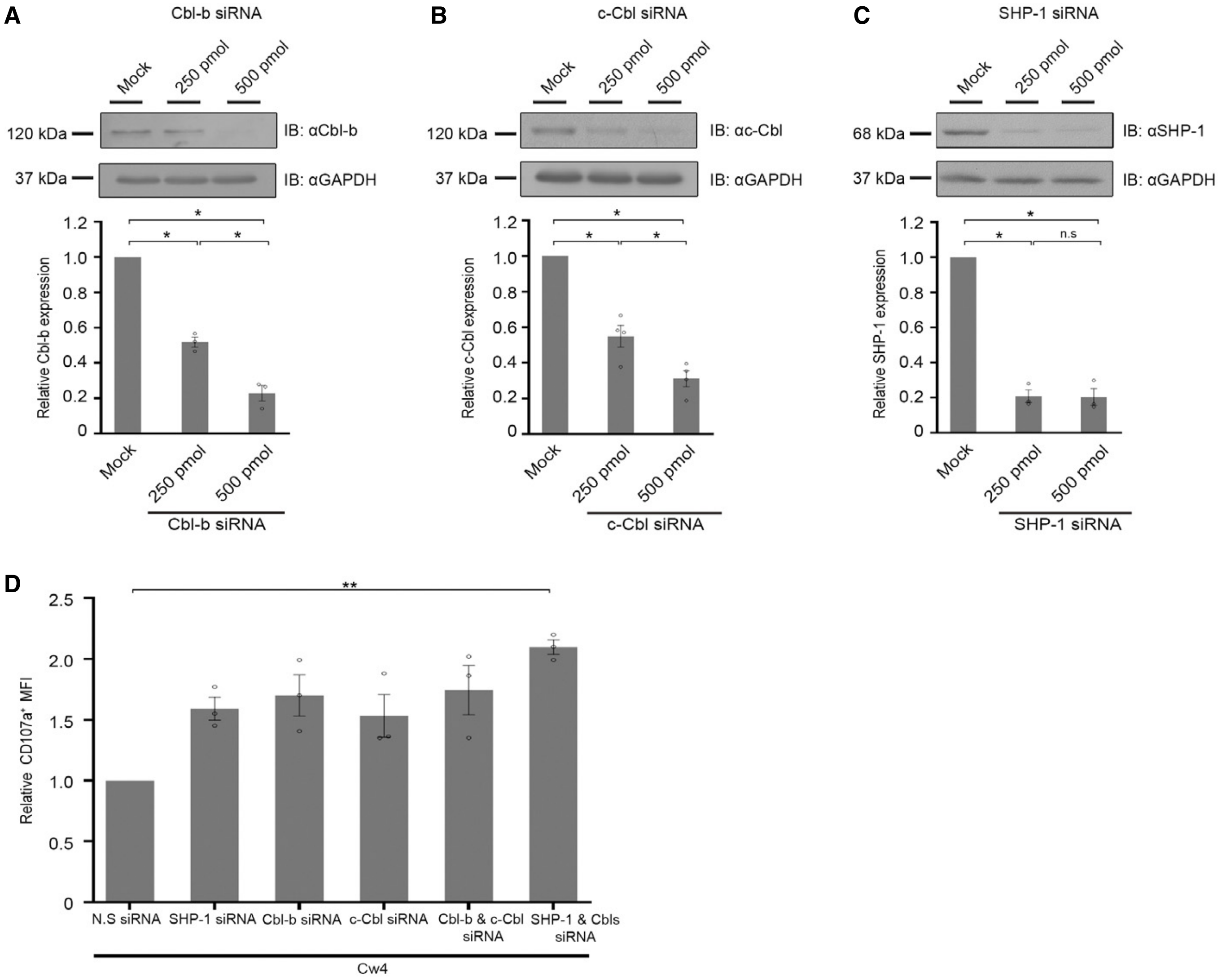

Figure EV1. Gene silencing of SHP-1 and Cbls enhances NK cell function.

A–C YTS-2DL1 cells were either mock-transfected or transfected with 250 or 500 pmol of (A) Cbl-b siRNA, (B) c-Cbl siRNA, or (C) SHP-1 siRNA using Amaxa electroporation. After 48 h, cells were lysed, and the nitrocellulose membranes were blotted with anti-Cbl-b, anti-c-Cbl, or anti-SHP-1 antibodies. GAPDH served as a loading control. Densitometric analysis of the bands was performed using ImageJ and normalized to the GAPDH densitometry values. Relative expression of the three proteins compared to the mock control group is presented within the graph. Analysis by ImageJ densitometry revealed a decrease of Cbl-b siRNA: 48 ± 3% and 77 ± 4%, $P \leq 0.03$ for Cbl-b, 45 ± 6% and 68 ± 4%, $P \leq 0.03$ for c-Cbl, and 79 ± 3% and 79 ± 5%, $P \leq 0.03$ for SHP-1 following siRNA gene silencing concentrations of 250 pmol and 500 pmol, respectively (shown in bar graphs underneath each blot). The data represent three independent experiments ($n = 3$). Data are shown as mean ± SEM. *P* values were calculated vs mock-treated control cells by one-sample *t*-tests and independent *t*-test. *P* values are indicated by asterisks. $^{*}P \leq 0.05$.

D YTS KIR2DL1 cells were gene silenced for either SHP-1, Cbl-b, c-Cbl or a combination of both Cbls proteins or of Cbls and Shp-1. NK cells treated with N.S siRNA served as control. After 48 h, YTS KIR2DL1 were incubated with 721.221 Cw4 target cells for 2 h and analyzed by flow cytometry to determine the expression of CD107a. Expression of CD107a was compared by mean fluorescence intensity (MFI). Relative expression of CD107a MFI was normalized to the mock-transfected sample following Cw4 incubation. The data represent three independent experiments ($n = 3$). Data are shown as mean ± SEM. *P* values were calculated by one-sample *t*-test and are indicated by asterisks. $^{**}P \leq 0.001$.

Data information: Exact *P* values are shown in Appendix Table S1.

© 2022 The Authors

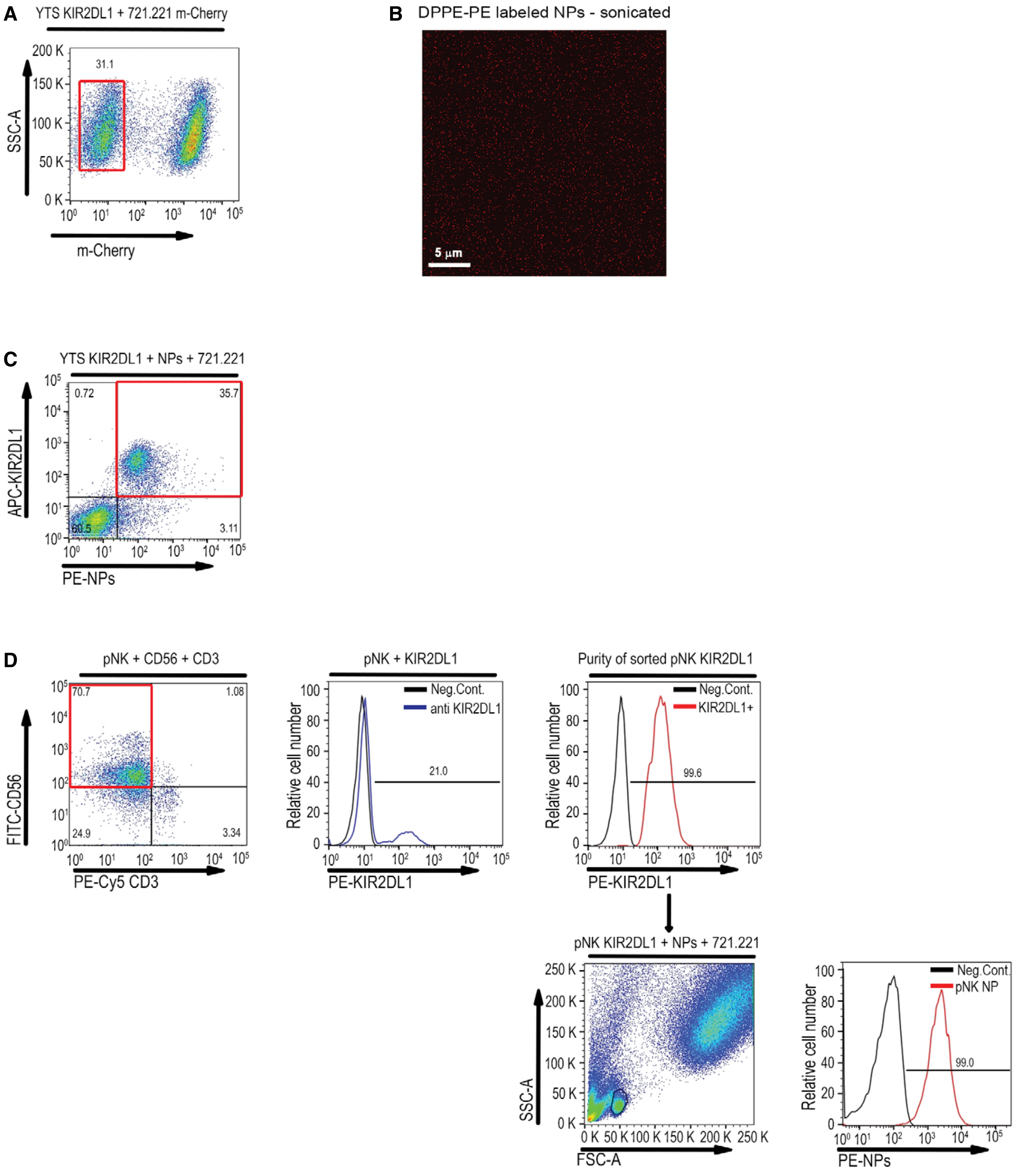

Figure EV2.

© 2022 The Authors

Figure EV2. Gating strategies for the identification of NK cells.

A Gating strategy to distinguish between YTS-2DL1 cells and 221-Cw4/7-expressing mCherry cells, using side scatter (SSC) and mCherry. Cells that were negative for mCherry were analyzed for CD107a expression.
B Imaging of fluorescently labeled NPs using confocal microscopy following sonication. The data shown are representative of three independent experiments.
C Gating strategy to define YTS-2DL1 incorporated NKp46 antibody-coated NPs population, using anti-KIR2DL1 to define NK cells. Cells that were positive for both rhodamine-labeled NPs and KIR2DL1 antibody staining were analyzed for CD107a expression.
D Primary NK cells were stained with PE-Cy5-CD3 and FITC-CD56 antibodies followed by staining with PE-KIR2DL1/S1. The pNK-expressing KIR2DL1 subset was then enriched by FACS sorting according to the PE signal. This subset was then incubated with target cells. Gating strategy to define the pNK-KIR2DL1^{+} using forward scatter (FSC) and side scatter (SSC). Cells that were positive for rhodamine-labeled NPs were analyzed for CD107a expression.

Figure EV3. *In vivo* toxicity and biodistribution of NKp46 antibody-coated NPs in tumor-bearing NRG mice following efficacy study end point.

A Diabetic/severe combined immunodeficiency (SCID/NOD) mice received intravenous (I.V) injection of NKp46 NPs every 72 h for a total of six injections. Mice were monitored for weight loss daily over the course of 23 days following the first treatment. Mice were treated with NK cells' gene silenced for SHP-1 and Cbls using siRNA-loaded NPs (n = 13) or N.S siRNA-loaded NPs (n = 13) for three independent experiments.
B Mice were I.V administered fluorescently labeld NKp46-NPs, or PBS as negative control. Mice were euthanized at indicated times following I.V injection of the NKp46-NPs. Internal organs and tumors were taken out and imaged using computed tomography, CRi Maestro II.
C Calculation of average rhodamine fluorescence signal (10^6 photon/cm^2/s) per organ 24, 48, and 72 h from injection was performed using the Maestro software.

© 2022 The Authors

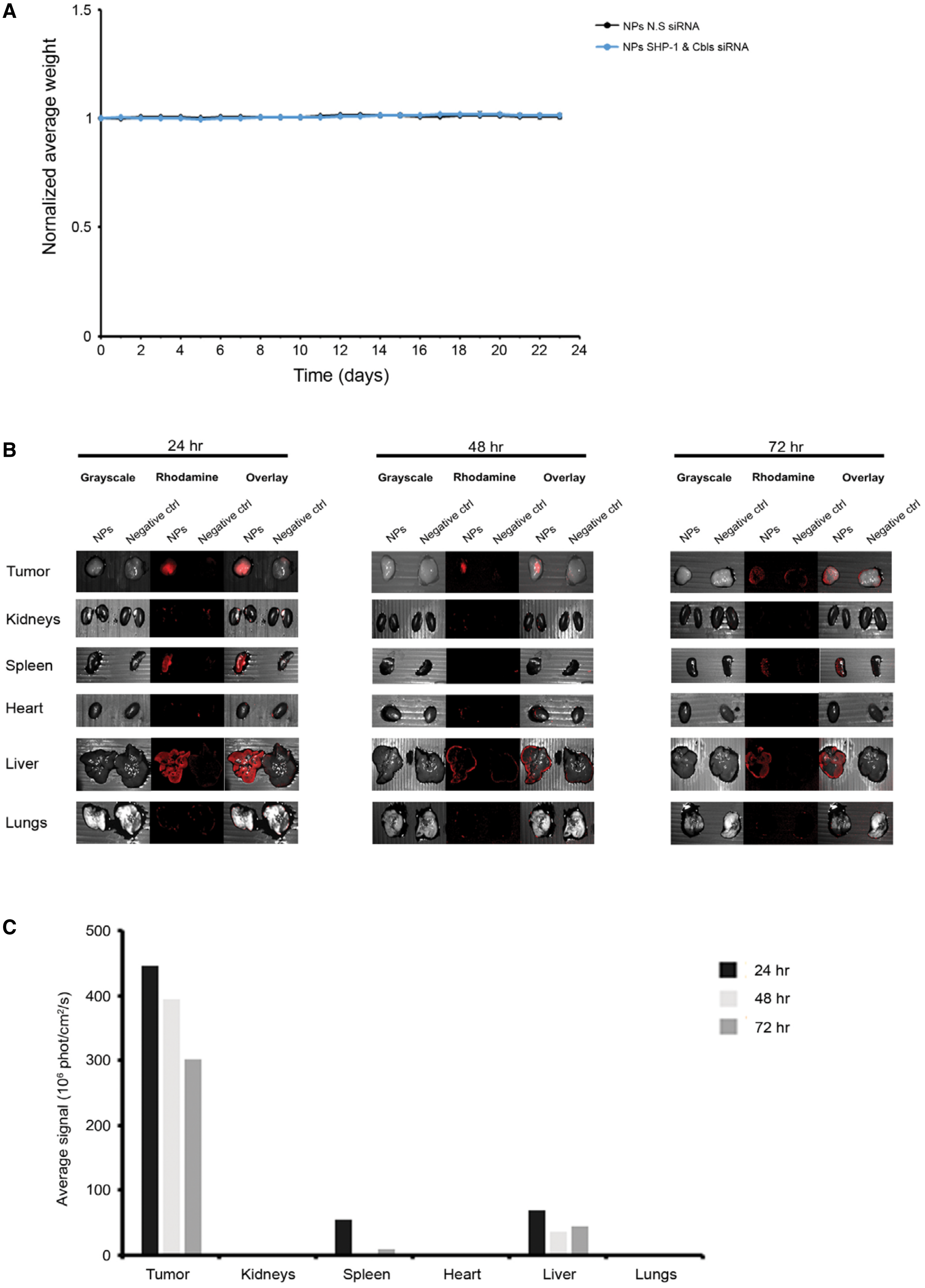

Figure EV3.

 © 2022 The Authors

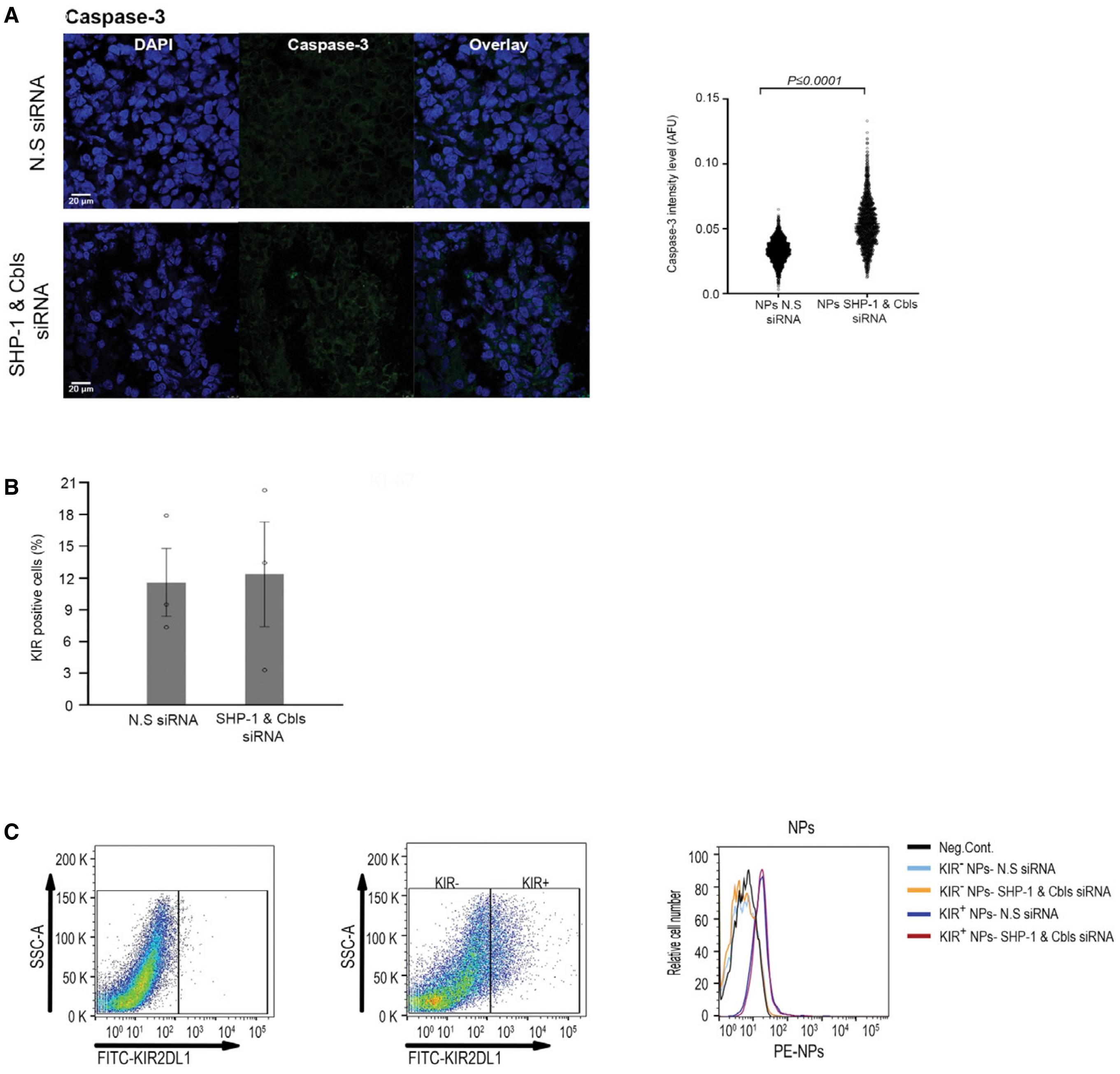

Figure EV4. Anti-tumor activity and activation of tumor-infiltrating NK cells.

A IHC analysis of apoptotic tumor cells via cleaved Caspase-3 staining of tumor sections from NS (n = 1,875 cells) vs SHP-1 and Cbls NPs (n = 1,477 cells) treated mice. P value was calculated by two-tailed Student's t-test and indicated within the graph $P \leq 0.0001$.

B KIR expression on dissociated tumors to detect NK cells. Data are means ± SEM of three independent experiments (n = 3).

C FACS analysis of disassociated tumors from NS vs SHP-1 and Cbls NPs treated mice. NK cells were identified and gated from single-cell suspensions by staining for KIR2DL1, and analyzed for the percentage of NK cells incorporating fluorescently labeled NPs.

Data information: Exact P values are shown in Appendix Table S1.

Source data are available online for this figure

© 2022 The Authors
