# Supplementary figures and images for: Modulation of intrinsic inhibitory checkpoints using nano‐carriers to unleash NK cell activity
Source: EMBO Mol Med. 2021 Nov 2;14(1):e14073. doi: 10.15252/emmm.202114073 (PMC8749471; doi:10.15252/emmm.202114073)

Source Data Figure 1

A

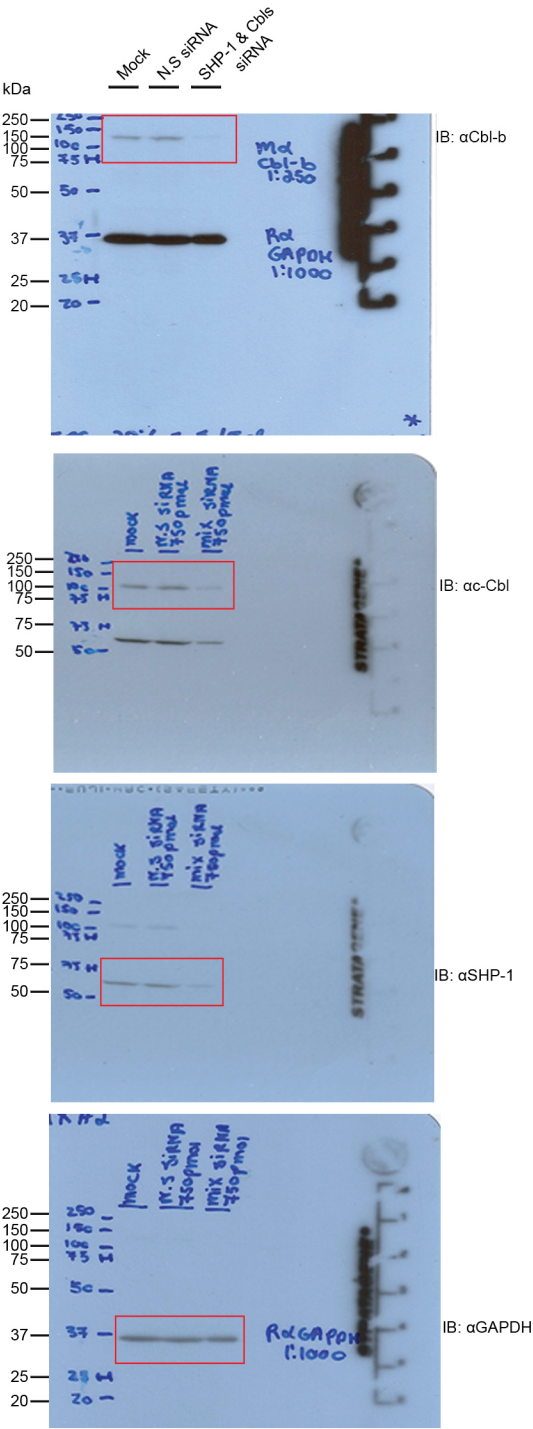

Supplement: Supplementary file 4 — Source Data for Figure 1 [file EMMM-14-e14073-s005.zip › Figure_1/WB_source_data_Figure_1A.pdf]

Source Data Figure 4

A

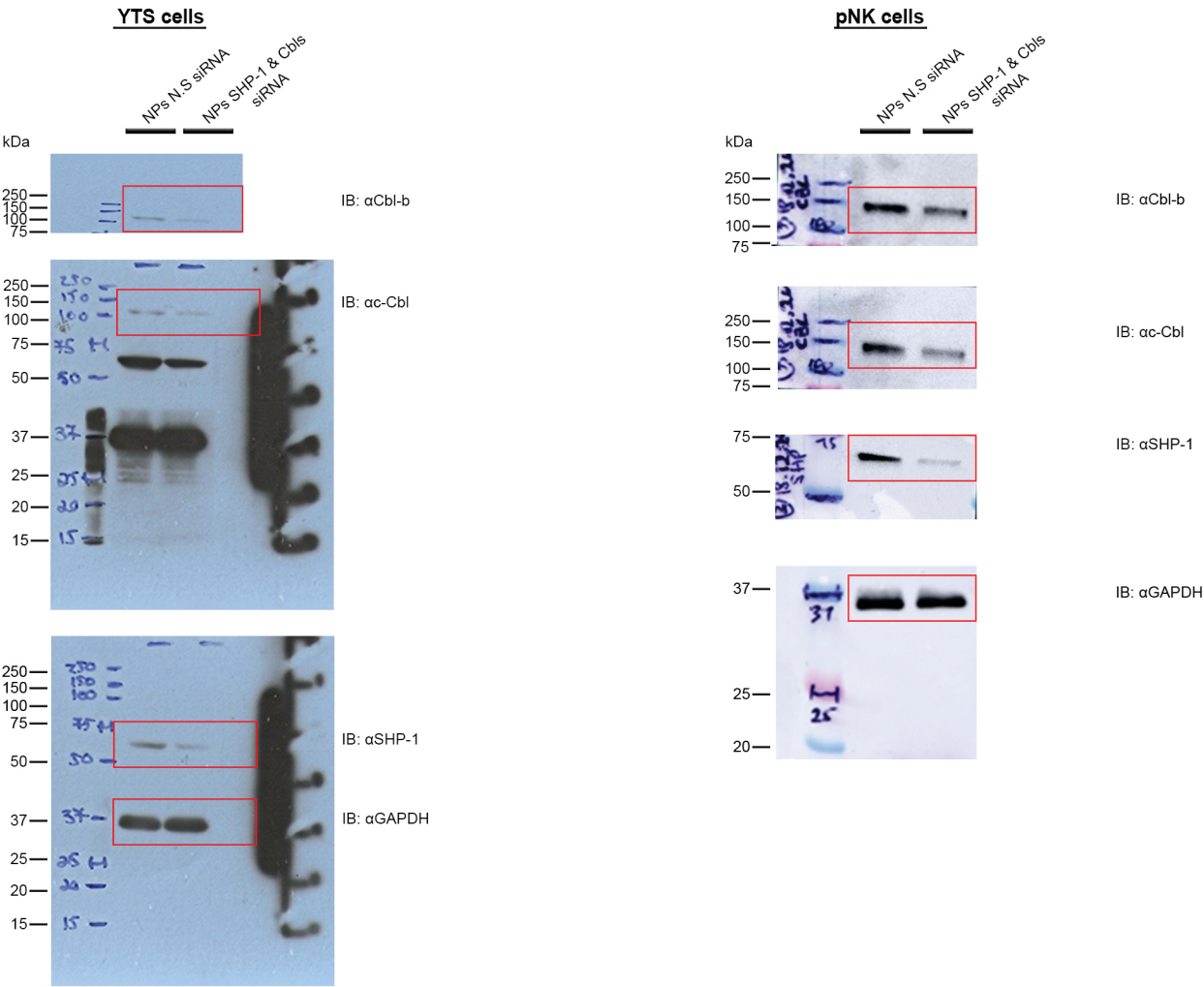

Supplement: Supplementary file 5 — Source Data for Figure 4 [file EMMM-14-e14073-s001.zip › Figure_4/WB_source_data_Figure_4A.pdf]
