# Supplementary material for: Modulation of intrinsic inhibitory checkpoints using nano‐carriers to unleash NK cell activity
Source: EMBO Mol Med. 2021 Nov 2;14(1):e14073. doi: 10.15252/emmm.202114073 (PMC8749471; doi:10.15252/emmm.202114073)
Supplement: Supplementary file 8 [file EMMM-14-e14073-s006.pdf]

EMBO Molecular Medicine

Modulation of intrinsic inhibitory checkpoints using nanocarriers to unleash NK cell activity

Guy Biber, Batel Sabag, Anat Raiff, Aviad Ben-Shmuel, Abhishek Puthenveetil, Jennifer Benichou, Tammir Jubany, Moria Levy, Shiran Killner, and Mira Barda-Saad
DOI: 10.15252/emmm.202114073

Corresponding authors: Mira Barda-Saad (Mira.Barda-Saad@biu.ac.il) , Jennifer Benichou (jeniiii.bee@gmail.com)

Review Timeline:

Submission Date:	3rd Feb 21
Editorial Decision:	1st Mar 21
Revision Received:	29th Jun 21
Editorial Decision:	20th Jul 21
Revision Received:	29th Jul 21
Editorial Decision:	31st Aug 21
Revision Received:	22nd Sep 21
Accepted:	30th Sep 21

Editor: Lise Roth

Transaction Report:

(Note: With the exception of the correction of typographical or spelling errors that could be a source of ambiguity, letters and reports are not edited. Depending on transfer agreements, referee reports obtained elsewhere may or may not be included in this compilation. Referee reports are anonymous unless the Referee chooses to sign their reports.)

Dear Prof. Barda-Saad,

Thank you for submitting your manuscript (EMBOJ-2021-107698) to The EMBO Journal. I have now had a chance to read it carefully and to discuss it with my colleagues.

Your analysis reports on the development of a novel therapeutic approach to interfere with NK cell activation. The system is based upon using lipid-based nano-carriers containing siRNAs against SHP-1 and Cbls that are targeting to NK cells using a monoclonal anti-NKp46 antibody. The findings show that the developed system works well and that it targets NK cells, block SHP-1 and Cbls expression leading to NK activation and that this blocks tumor growth in mice. I appreciate the potential therapeutic implications of the reported and see the value if the developed system. However, I am also afraid that scope wise that we find that the manuscript is not a good fit for consideration in The EMBO Journal as we gain limited novel insight NK cell activation or how that affects tumour growth.

Given the potential therapeutic implications of the findings, I have taken the opportunity to discuss the manuscript with my colleagues at EMBO Molecular Medicine (EMM). EMM is interested in considering the manuscript for publication and will send the study out for full review if you submit to them. You can choose the link below to do so.

For The EMBO Journal I am sorry for not having more positive news on this occasion, but I hope that you will consider the EMM Option.

with best wishes

Karin

Karin Dumstrei, PhD
Senior Editor
The EMBO Journal

1st Mar 2021

Dear Prof. Barda-Saad,

Thank you for the submission of your manuscript to EMBO Molecular Medicine. We have now received feedback from two of the three reviewers who agreed to evaluate your manuscript. Given that referee #1 has unfortunately not returned his/her report so far, and that both referees 2 and 3 provide similar recommendation, we prefer to make a decision now in order to avoid further delay in the process. Should referee #1 provide a report, we will send it to you, with the understanding that we would not ask you for extensive experiments in addition to the ones required in the enclosed reviews.

As you will see from the reports below, the referees acknowledge the interest of the study and are overall supporting publication of your work pending appropriate revisions. Addressing the reviewers' concerns in full will be necessary for further considering the manuscript in our journal. However, upon cross-commenting with the referees, we also agreed that the assessment of toxicity and off-targets effects (referee #2 point #2) in an adequate in vivo would not be required, but should at least be discussed.

Acceptance of the manuscript will entail a second round of review. EMBO Molecular Medicine encourages a single round of revision only and therefore, acceptance or rejection of the manuscript will depend on the completeness of your responses included in the next, final version of the manuscript. For this reason, and to save you from any frustrations in the end, I would strongly advise against returning an incomplete revision.

When submitting your revised manuscript, please carefully review the instructions that follow below. Failure to include requested items will delay the evaluation of your revision:

1) A .docx formatted version of the manuscript text (including legends for main figures, EV figures and tables). Please make sure that the changes are highlighted to be clearly visible.

2) Individual production quality figure files as .eps, .tif, .jpg (one file per figure).

3) A .docx formatted letter INCLUDING the reviewers' reports and your detailed point-by-point responses to their comments. As part of the EMBO Press transparent editorial process, the point-by-point response is part of the Review Process File (RPF), which will be published alongside your paper.

4) A complete author checklist, which you can download from our author guidelines (https://www.embopress.org/page/journal/17574684/authorguide#submissionofrevisions). Please insert information in the checklist that is also reflected in the manuscript. The completed author checklist will also be part of the RPF.

5) We would also encourage you to include the source data for figure panels that show essential data. Numerical data should be provided as individual .xls or .csv files (including a tab describing the data). For blots or microscopy, uncropped images should be submitted (using a zip archive if multiple images need to be supplied for one panel). Additional information on source data and instruction on how to label the files are available at
.

6) Our journal encourages inclusion of *data citations in the reference list* to directly cite datasets that were re-used and obtained from public databases. Data citations in the article text are distinct from normal bibliographical citations and should directly link to the database records from which the data can be accessed. In the main text, data citations are formatted as follows: "Data ref: Smith et al, 2001" or "Data ref: NCBI Sequence Read Archive PRJNA342805, 2017". In the Reference list, data citations must be labeled with "[DATASET]". A data reference must provide the database name, accession number/identifiers and a resolvable link to the landing page from which the data can be accessed at the end of the reference. Further instructions are available at .

7) We replaced Supplementary Information with Expanded View (EV) Figures and Tables that are collapsible/expandable online. A maximum of 5 EV Figures can be typeset. EV Figures should be cited as 'Figure EV1, Figure EV2" etc... in the text and their respective legends should be included in the main text after the legends of regular figures.

- For the figures that you do NOT wish to display as Expanded View figures, they should be bundled together with their legends in a single PDF file called *Appendix*, which should start with a short Table of Content. Appendix figures should be referred to in the main text as: "Appendix Figure S1, Appendix Figure S2" etc.

- Additional Tables/Datasets should be labeled and referred to as Table EV1, Dataset EV1, etc. Legends have to be provided in a separate tab in case of .xls files. Alternatively, the legend can be supplied as a separate text file (README) and zipped together with the Table/Dataset file.
See detailed instructions here:
.

8) The paper explained: EMBO Molecular Medicine articles are accompanied by a summary of the articles to emphasize the major findings in the paper and their medical implications for the non-specialist reader. Please provide a draft summary of your article highlighting
- the medical issue you are addressing,
- the results obtained and
- their clinical impact.

This may be edited to ensure that readers understand the significance and context of the research. Please refer to any of our published articles for an example.

9) For more information: There is space at the end of each article to list relevant web links for further consultation by our readers. Could you identify some relevant ones and provide such information as well? Some examples are patient associations, relevant databases, OMIM/proteins/genes links, author's websites, etc...

10) Every published paper now includes a 'Synopsis' to further enhance discoverability. Synopses are displayed on the journal webpage and are freely accessible to all readers. They include a short stand first (maximum of 300 characters, including space) as well as 2-5 one-sentences bullet points that summarizes the paper. Please write the bullet points to summarize the key NEW findings. They should be designed to be complementary to the abstract - i.e. not repeat the same text. We encourage inclusion of key acronyms and quantitative information (maximum of 30 words / bullet point). Please use the passive voice. Please attach these in a separate file or send them by email, we will incorporate them accordingly.

Please also suggest a striking image or visual abstract to illustrate your article. If you do please provide a png file 550 px-wide x 400-px high.

11) As part of the EMBO Publications transparent editorial process initiative (see our Editorial at http://embomolmed.embopress.org/content/2/9/329), EMBO Molecular Medicine will publish online a Review Process File (RPF) to accompany accepted manuscripts.
In the event of acceptance, this file will be published in conjunction with your paper and will include the anonymous referee reports, your point-by-point response and all pertinent correspondence relating to the manuscript. Let us know whether you agree with the publication of the RPF and as here, if you want to remove or not any figures from it prior to publication.
Please note that the Authors checklist will be published at the end of the RPF.

EMBO Molecular Medicine has a "scooping protection" policy, whereby similar findings that are published by others during review or revision are not a criterion for rejection. Should you decide to submit a revised version, I do ask that you get in touch after three months if you have not completed it, to update us on the status.

I look forward to receiving your revised manuscript.

Yours sincerely,

Lise Roth

Lise Roth, PhD
Editor
EMBO Molecular Medicine

***** Reviewer's comments *****

Referee #2 (Comments on Novelty/Model System for Author):

The in vivo model's relevance is weakened by using human tumour and NK cell lines in immunocompromised mice, however, if these limitations were clearly acknowledged it might be adequate, see comments below.

Referee #2 (Remarks for Author):

The authors propose tuning the NK cell activation threshold using siRNA delivered by liposomes coated with anti-NKp46 as a potential NK cell-directed immunotherapy. While the findings are interesting and the paper mostly clearly presented, there are important overstatements considering the limitations of the models used. The points below should be addressed to strengthen the paper:

Major points

-The specificity of the anti-NKp46 nanoparticles was addressed using specific cell lines rather than whole blood. What about highly phagocytic cells? Showing by flow cytometry of blood that only NK cells and not neutrophils or monocytes take them up, is an important control (especially considering that even though the mean NP size looks small in Fig2B there seems to be much larger clusters in Fig2C).
-Importantly related to the need to address the point above, in the in vivo experiments human siRNA, a human NK cell and tumour cell line in immunocompromised mice are used, so this is unlikely to pinpoint any potential toxicity or off target effects.
-The in vivo experiment does not address the proposed use of this type of therapy as 3x10^6 YTS cells are injected intratumorally. What would happen if the YTS cells were transferred i.v. or IP? If the therapy only works when there are high numbers of NK cells infiltrating the tumour, this is still potentially exciting, but this should be properly discussed in the text.
-Is expression or function of NKp46 affected upon interaction with the anti-NKp46 nanoparticles?
-Hyaluronan coating is suggested to increase the accumulation of the NPs at the tumour site, but there are no data supporting this in the results.

Other points
-Fig EV1D does not provide evidence that there is a synergistic effect. It looks more like an additive effect. Moreover, the c-Cbl siRNA seems redundant.
-Fig 1B legend: typo the curve is blue not green.
-In all figures, "more than 3 independent experiments" is stated, the number of experiments in each group should be more precisely described and individual data points shown in the graphs (perhaps by superimposing them on the bars).
-In some cases only representative plots are shown without cumulative data, and, on the other hand, for CD107a cumulative data are shown but no representative histogram or dot plot is displayed. Was the MFI taken on gated NK cells? It is not clear from the M&M as no other Ab was stated as being used in this assay.
-What hybridoma was used for anti-NKp46 production?

Referee #3 (Comments on Novelty/Model System for Author):

Technical quality is medium because the authors did not use the proper statistical tests (detailed to them).
The novelty and medical impact is high because the development of nanoparticles that are targeted to specific cell subsets is an issue properly addressed in the manuscript.
The model is adequate because, based on the data presented, there is a possible/future translation of the findings to the treatment of cancer.

Referee #3 (Remarks for Author):

In this manuscript, Biber G and coworkers explored how the knock-down of intracellular inhibitory molecules using nanocarriers unleashes NK cell activity. The authors describe a non-viral lipid nanoparticle-based delivery system that encapsulates siRNA to silence genes that encode for critical intracellular inhibitory NK cell molecules such as SHP-1, c-Cbl, and Cbl-b, and demonstrate

that these nanoparticles efficiently and specifically target NK cells in-vivo, silence inhibitory checkpoint signaling molecules, and unleash NK cell activity to eliminate tumors. Finally, the authors claim that this study demonstrates that nanoparticles encapsulating small molecules constitute an effective systemic in-vivo delivery strategy to enhance NK cytotoxicity in the tumor microenvironment. The study has been well designed and provides interesting information. However, there are several aspects that they must address to improve the manuscript.

Major comments

It is not clear why the authors determined the impact of SHP-1 and Cbl silencing of NK cell activity using target cells that over-express HLA molecules that engage KIR receptors. To test this idea, experiments using NK cells activated with target cells that express/over-express ligands for activating receptors and/or stimulation with cytokines such as IL-12 and IL-18 would provide more solid evidence. THi is because NK cells usually become activated in a tumor niche mostly due to upregulation of ligands for activation receptors on tumor cells. As performed, the experiment only leads to the conclusion that "Cbl-b, c-Cbl, and SHP-1 regulate the NK cell activation threshold" when inhibitory KIR are simultaneously engaged, which is an already known phenomenon. As there are other inhibitory receptors that determine the NK cell activation threshold and activation receptors that must be engaged to stimulate NK cell activation, the results of the experiments with HLA-Cw4-expressing cells are not convincing and the conclusion that "these inhibitory checkpoint molecules are attractive targets for increasing NK cell function" should be sustained with more convincing experimental evidence.

Statistical analysis in Figures 1, 4, 5 and 6 are not correct. t-test can only be applied if there are two experimental groups. Please, make the necessary corrections to the statistical test used. Figure 6F requires the use of a log rank test.

Figures 1C, 4C, 4F and 6E. The relative MFI of CD107a expression were depicted. However, it is better to show the actual values of MFI instead of the relative values.

Figure 6. The authors describe that they injected human NK cells in B cell lymphoma-engrafted mice and then, mice were treated with the NP. However, as they tested the effect of HLA-Cw4 and HAL-Cw7 expression of KIR-mediated effects, KIR matching between donor of NK cells and B cell-lymphoma cells should be informed. This is because it is not clear if NK cells injected into the mice are subjected to KIR-mediated inhibitory effects that involve the SHP-1 and Cbl, or if the effect is mediated through other NK cell receptors.

Figure EV4C. To identify NK cells, the authors performed a tumor dissociation and flow cytometry analysis. However, as this is a complex sample, it is critical that the authors provide the gating strategy used to identify such NK cells. Also, as the gating strategy was not provided, it is curious that the histogram depicted in panel C is monomodal as one expects to have a sample that contains many different cells (tumor cells, NK cells, stromal cells).

Figure EV4D. The authors describe that "NK cells from mice treated with SHP-1 and Cbl specific NP showed a decrease in the inhibitory receptor, Tigit, relative to NK cells from mice treated with NPs encapsulating NS siRNAs". First, the data presented do not show decreased expression. Presenting percentages in the histogram, the data show changes in the frequencies of positive cells. Second, presenting data from only one animal does not indicate that this is a general phenomenon that occurs in all mice. Third, the origin and specificity of the anti-TIGIT mAb was not specified. And finally, the rationale of staining TIGIT+ cells within the tumor were not explained and, as presented, these data are confusing.

The strategy of delivering nanoparticles is interesting. However, to be of wide interest, one important aspect is the duration of the effect. In figure 6, the authors use a protocol in which they injected NK cells 6 times and treated with the nanoparticles 6 times (always 24 h after the injection of the NK cells). However, it is important to establish whether NK cells persist and for how long. Is it necessary to inject NK cells every 3 days? How long do NK cells persist? Perhaps, it is only necessary to reinject the particles with certain frequency and it is not necessary to reinject NK cells.

Minor criticisms

Please, specify the name of the clone and origin of the anti-NKp46 mAb (mentioned on page 27).

The analysis of cytokines produced by NK cells (or PBMC) treated with different nanoparticles should be renamed as it does not constitute a "cytokine storm" as mentioned by the authors (page 27).

Silencing of c-Cbl shown in Figure EV1B is barely visible. Please, provide an image where the differences are more visible.

TIGIT should be written in capital letters.

Reviewer's comments

Referee #2

Major points

1.a.-The specificity of the anti-NKp46 nanoparticles was addressed using specific cell lines rather than whole blood. What about highly phagocytic cells? Showing by flow cytometry of blood that only NK cells and not neutrophils or monocytes take them up, is an important control (especially considering that even though the mean NP size looks small in Fig2B there seems to be much larger clusters in Fig2C).

As requested by the reviewer, we added an experiment in which we treated whole blood with NKp46-coated NPs and differentiated between the cell populations according to their volume, density and CD45 expression level (Figure R1-left and middle panels). As shown, NKp46-coated NPs are incorporated into lymphocytes and also into monocytes and granulocytes but only to a small extent. However, while the incorporation into the primary NK cells (pNKs) is in proportion to the expression of anti-NKp46 (Fig 3A, B), the incorporation of the NPs to neutrophils and macrophages is non-specific and is based on the chemical features of the NPs. It was already demonstrated that under the shear force of blood flow, the neutrophils and monocytes exhibit reduced interaction with the NPs, potentially due to detachment of the NPs bound by weak cellular interactions; these forces are not sufficient to reduce the stronger antigen-specific interaction as occurs in pNKs (Vu et al. 2020).
Furthermore, we would like to bring to the referee's attention, Figure EV4C (manuscript and Figure R1-right panel), demonstrating that indeed in our in-vivo experiments, NKp46-coated NPs are specifically integrated by the KIR-expressing cells extracted from mice bearing tumors, and not by the KIR negative cells, demonstrating that in-vivo the incorporation of the NPs to non-NK cells is negligible.

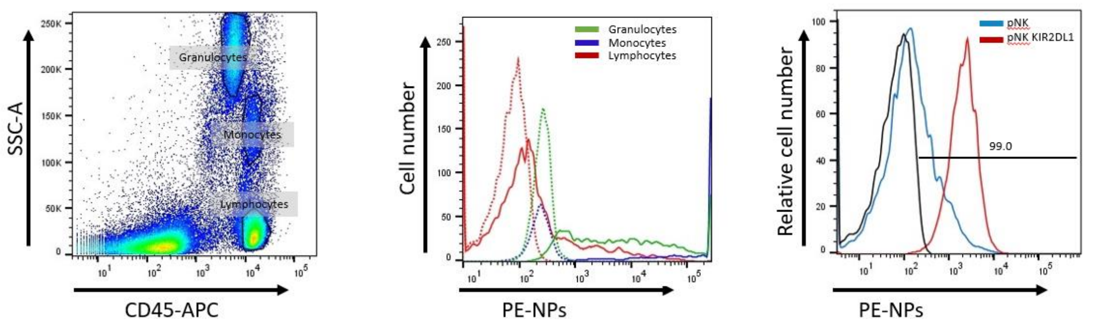

Figure R1: Whole blood cells were differentiated by cell's granularity vs. CD45 expression to cell subsets including granulocytes, monocytes, and lymphocytes following their incubation with NKp46-coated NPs. Dashed line-Untreated cells; Continues line-Cells treated with coated NPs.

1.b.- The mean NP size looks small in Fig2B there seems to be much larger clusters in Fig2C.

According to dynamic light scattering (DLS) measurements, the zeta potential of our NPs (-24.1±2.9 mV, Figure 2B) indicates that they have sufficient repulsive

force to attain physical colloidal stability, thus preventing their aggregation. We think that the clustering of NPs seen in Figure 3B was not due to particle aggregation, but rather is due to saturated fluorescence intensity or different Z stacks. To address this point, we now provide new images including a better resolved confocal image with fluorescence intensity adjustments (Figure 2C). Furthermore, to confirm that aggregation does not occur, we included an image of NPs subjected to sonication. The NPs in both the sonicated and control samples showed similar size and fluorescence intensity further supporting the lack of NP aggregation.

2.-Importantly related to the need to address the point above, in the in vivo experiments human siRNA, a human NK cell and tumour cell line in immunocompromised mice are used, so this is unlikely to pinpoint any potential toxicity or off target effects.

The text was changed based on this comment by the referee. Indeed, the use of a humanized mouse model would allow us to better rule out potential toxicity or off target effects; however such a study is beyond the scope of this paper. We wish to emphasize that the mice used in these experiments did not show any stress-related side effects during NP treatments. Moreover, the use of NPs had no effect on mouse weight (mouse body score index) (Figure EV3A) nor on NK cellular activation as determined by calcium release and cytokine secretion (Figure 5).

3-The in vivo experiment does not address the proposed use of this type of therapy as 3x10^6 YTS cells are injected intratumorally. What would happen if the YTS cells were transferred i.v. or IP? If the therapy only works when there are high numbers of NK cells infiltrating the tumour, this is still potentially exciting, but this should be properly discussed in the text.

We thank the reviewer for this comment and we now address this issue in the Discussion (see page 23, paragraph 8). The goal of this study was to determine the potential of using NPs to target NK intracellular inhibitory check points with the goal of enhancing NK cell activation and mitigating tumor growth in-vivo. Thus, by injecting a constant number of YTS intratumorally (based on Töpfer et al. 2015), we determined the potential of manipulating NK cell behavior and activity by the NPs (injected intravenously). It is important to mention that despite it being an interesting issue in itself, in this study, we did not focus on NK intravasation to the tumor microenvironment (TME) which was already extensively studied and analyzed by others (Vitale et al. 2014).

4-Is expression or function of NKp46 affected upon interaction with the anti-NKp46 nanoparticles?

The anti-NKp46 antibody we used does not affect either NK activation or function (please see Shemer-Avni et al. 2017) Also, Figure 5A demonstrates that NK cell incubation with anti-NKp46 antibody-coated NPs (green curve) does not cause any increase in intracellular calcium flux, indicating that the antibody itself does not induce NK activation.

5-Hyaluronan coating is suggested to increase the accumulation of the NPs at the tumour site, but there are no data supporting this in the results.

As previously demonstrated by Bachar G et al (Bachar et al. 2011), Hyaluronan coating does not increase the accumulation of the NPs at the tumor site, but rather (1) serves as an efficient anchor for the antibody, (2) improves the half-life of the NPs in the circulation by providing a 'stealth' coating layer on the NP surface, enabling them to escape from recognition and clearance of phagocytic cells. We now added a paragraph to the Discussion describing the role of hyaluronan within the NPs (see page 21, paragraph 7).

Other points

1-Fig EV1D does not provide evidence that there is a synergistic effect. It looks more like an additive effect. Moreover, the c-Cbl siRNA seems redundant.

The text was corrected based on the reviewer's comment.

2-Fig 1B legend: typo the curve is blue not green.

The legend was corrected accordingly.

3-In all figures, "more than 3 independent experiments" is stated, the number of experiments in each group should be more precisely described and individual data points shown in the graphs (perhaps by superimposing them on the bars).

Legends and graphs were modified, as suggested by the reviewer to more specifically show the results of replicate experiments.

4-In some cases only representative plots are shown without cumulative data, and, on the other hand, for CD107a cumulative data are shown but no representative histogram or dot plot is displayed. Was the MFI taken on gated NK cells? It is not clear from the M&M as no other Ab was stated as being used in this assay.

Two gating strategies were used: For the experiments with YTS-incorporated NPs, the gates were set based on viability, as Cw4 express neither NKp46 nor KIR2DL1 receptor, and therefore were not subjected to NKp46-coated NP uptake (demonstrated in Figure 3A-B). For the experiments in Figure 1 and EV1, fluorescently labelled target cells were distinguished from the YTS cells. The gating strategy is now presented in Figure EV2A, C.
In the experiments performed on pNKs, the gate was set based on cell's volume vs. granularity, as pNKs can be clearly distinguished from Cw4 according to their size.
Of note, primary NK cells were isolated from PBMCs of healthy donors using the EasySep™ human NK cell enrichment kit, following sorting based on CD3 CD56 and KIR2DL1 expression.

The gating strategy is now presented in Figure EV2D.

5-What hybridoma was used for anti-NKp46 production?

The hybridoma used was D2-9A5, specific to the D2 domain of NKp46 as previously described in detail (Mandelboim et al. 2001; Shemer-Avni et al. 2017).

Referee #3:

Major comments:

1. It is not clear why the authors determined the impact of SHP-1 and Cbl silencing of NK cell activity using target cells that over-express HLA molecules that engage KIR receptors. To test this idea, experiments using NK cells activated with target cells that express/over-express ligands for activating receptors and/or stimulation with cytokines such as IL-12 and IL-18 would provide more solid evidence. This is because NK cells usually become activated in a tumor niche mostly due to upregulation of ligands for activation receptors on tumor cells.
As performed, the experiment only leads to the conclusion that "Cbl-b, c-Cbl, and SHP-1 regulate the NK cell activation threshold" when inhibitory KIR are simultaneously engaged, which is an already known phenomenon. As there are other inhibitory receptors that determine the NK cell activation threshold and activation receptors that must be engaged to stimulate NK cell activation, the results of the experiments with HLA-Cw4-expressing cells are not convincing and the conclusion that "these inhibitory checkpoint molecules are attractive targets for increasing NK cell function" should be sustained with more convincing experimental evidence.

In this study, we developed an approach to interfere with negative signaling cascades by inhibiting the key negative regulators, SHP-1 and Cbls, in order to upregulate the NK cell activation threshold and NK function under inhibitory conditions (Fig. 1C and Fig. 4C-F). The experimental system used was based on either YTS-KIR2DL1, an NK cell line which stably expresses KIR2DL1, or primary NK cells expressing KIR2DL1 (pNK KIR2DL1$^+$). Both cell types were incubated with two subsets of target cells, 721.221 cells, which express HLA of allotype Cw7, an irrelevant MHC class I molecule, or 721.221 cells, which express HLA of allotype Cw4, which is the ligand for the KIR2DL1 inhibitory receptor.
Tumor escape from NK cell immune surveillance is mainly regulated by two molecular mechanisms: 1. Reduction of activating signals by decreasing activating receptor ligand expression on cancer cells. 2. Increase in inhibitory signals delivered to NK cells by preserving the expression of HLAs on cancer cells (Ben-Shmuel A. et al. 2020). Our model system addresses the second mechanism. Furthermore, while most current strategies focus on inhibitory check point blockade (ICB) using antibodies to redundant surface receptors, we aim in our study to block key intracellular inhibitory signals common to most inhibitory receptors. Indeed, incubating YTS cells with 721.221 Cw7 target cells demonstrated no significance difference between NK cells gene silenced for SHP-

1 and Cbls vs. control cells treated with non-specific siRNA (see new Figure 4C *P*=0.38, new Figure 4F *P*=0.08), indicating that our approach is specific to inhibitory engagement.

2. Statistical analysis in Figures 1, 4, 5 and 6 are not correct. t-test can only be applied if there are two experimental groups. Please, make the necessary corrections to the statistical test used. Figure 6F requires the use of a log rank test.
Figures 1C, 4C, 4F and 6E. The relative MFI of CD107a expression were depicted. However, it is better to show the actual values of MFI instead of the relative values.

We thank the reviewer for this comment. Extensive statistical analyses were added to the revised manuscript under the guidance of a professional statistician.
As specified below, five types of statistical tests were used:

1. One-way ANOVA followed by a post hoc analysis to correct for multiple comparisons (Tukey) (Figures 1C-E, Figure 4D-E and 5B);
2. One way ANOVA (Tukey's post hoc) together with one sample t-tests (FDR correction) (4C, 4F);
3. One sample t-tests (Figures 1A, 4A and 6E)
4. Independent sample t-test together with one sample t-tests followed by FDR correction (Figures 6E, EV1A-D and EV4A).
5. Log-rank test (Figure 6F).

Relative mean fluorescence intensities (MFI) were presented rather than absolute values due to intrinsic differences in staining conditions and FACS settings between independent experiments. However, in each experiment, the values were normalized relative to NK cells pretreated with N.S siRNA-loaded NPs/Mock incubation with Cw4, and as demonstrated, the trend was maintained throughout.

3.-Figure 6. The authors describe that they injected human NK cells in B cell lymphoma-engrafted mice and then, mice were treated with the NP. However, as they tested the effect of HLA-Cw4 and HLA-Cw7 expression of KIR-mediated effects, KIR matching between donor of NK cells and B cell-lymphoma cells should be informed. This is because it is not clear if NK cells injected into the mice are subjected to KIR-mediated inhibitory effects that involve the SHP-1 and Cbl, or if the effect is mediated through other NK cell receptors.

Please see our answer above to point #1. After establishing our experimental model in-vitro, we interrogated the mechanism of action in-vivo, focusing on suppressive conditions (HLA-Cw4).

Furthermore, the engagement specificity between HLA-Cw4 and KIR2DL1 is well established and was demonstrated by (1) (Colonna et al. 1993), (2) (Fan, Long, and Wiley 2001), and (3)(Matalon et al. 2018).

4. Figure EV4C. To identify NK cells, the authors performed a tumor dissociation and flow cytometry analysis. However, as this is a complex sample, it is critical that the authors provide the gating strategy used to identify such NK cells. Also, as the gating strategy was not provided, it is curious that the histogram depicted in panel C is monomodal as one expects to have a sample that contains many different cells (tumor cells, NK cells, stromal cells).

As requested by the reviewer, we added the gating strategy to new Figure EV4C. Tumors were extracted, a cell suspension was prepared, and cells were stained with anti-KIR2DL1 antibody. Tumor cells were differentiated based on KIR2DL1 expression level and were analyzed for the relative number of cells incorporating fluorescently labeled NPs based on PE fluorescence. As demonstrated, only YTS KIR2DL1^{+} cells were positive for NP incorporation. Non-NK cells were found negative to NPs (Fig. EV4C).

5.-Figure EV4D. The authors describe that "NK cells from mice treated with SHP-1 and Cbl specific NP showed a decrease in the inhibitory receptor, Tigit, relative to NK cells from mice treated with NPs encapsulating NS siRNAs". First, the data presented do not show decreased expression. Presenting percentages in the histogram, the data show changes in the frequencies of positive cells. Second, presenting data from only one animal does not indicate that this is a general phenomenon that occurs in all mice. Third, the origin and specificity of the anti-TIGIT mAb was not specified. And finally, the rationale of staining TIGIT+ cells within the tumor were not explained and, as presented, these data are confusing.

Based on the reviewer's comment, we removed this section from the manuscript.

6.-The strategy of delivering nanoparticles is interesting. However, to be of wide interest, one important aspect is the duration of the effect. In figure 6, the authors use a protocol in which they injected NK cells 6 times and treated with the nanoparticles 6 times (always 24 h after the injection of the NK cells). However, it is important to establish whether NK cells persist and for how long. Is it necessary to inject NK cells every 3 days? How long do NK cells persist? Perhaps, it is only necessary to reinject the particles with certain frequency and it is not necessary to reinject NK cells.

The NK cell line injected was YTS, which can -itself- form tumors. In order to prevent these cells from becoming tumorigenic, YTS were irradiated prior to injection (as described in Materials & Methods). Since irradiation might limit cell survival, we injected fresh irradiated cells every 72 hours. Note as well that our experimental approach was based on the already optimized technology published by Töpfer K. et al. (Töpfer et al. 2015).

Minor criticisms

1. Please, specify the name of the clone and origin of the anti-NKp46 mAb

The hybridoma used, D2-9A5, is specific to the D2 domain of NKp46, as was previously described in detail (Mandelboim et al. 2001; Shemer-Avni et al. 2017).

2. The analysis of cytokines produced by NK cells (or PBMC) treated with different nanoparticles should be renamed as it does not constitute a "cytokine storm" as mentioned by the authors (page 27).

We now use the term cytokine release or secretion.

3. Silencing of c-Cbl shown in Figure EV1B is barely visible. Please, provide an image where the differences are more visible.

New images are provided.

4.TIGIT should be written in capital letters.

We removed this section from the manuscript.

20th Jul 2021

Dear Prof. Barda-Saad,

Thank you for the submission of your revised manuscript to EMBO Molecular Medicine. We have now received feedback from the two referees who re-reviewed your manuscript.
As you will see from the reports below, while referee #3 is satisfied with the revisions, referee #2 still raises concerns that have not been addressed.
Therefore, we would like you to address the comments raised by this referee. Please be aware that this will be the last chance for you to address these points.

Additionally, please also address the following editorial issues:
- Please upload your figures in individual files
- Please provide an "Author contributions" section
- Please rename the "Conflict of interest" section
- Please reformat the references to have them in alphabetical order with 10 authors listed before et al.
- Please provide a 'The paper explained' section: EMBO Molecular Medicine articles are accompanied by a summary of the articles to emphasize the major findings in the paper and their medical implications for the non-specialist reader. Please provide a draft summary of your article highlighting:
- the medical issue you are addressing,
- the results obtained and
- their clinical impact.
- Thank you for providing a synopsis text and image. Please note that the text should include a short stand first (maximum of 300 characters, including space) as well as 2-5 one-sentences bullet points that summarizes the paper. Please write the bullet points to summarize the key NEW findings. They should be designed to be complementary to the abstract - i.e. not repeat the same text. We encourage inclusion of key acronyms and quantitative information (maximum of 30 words / bullet point). Please use the passive voice. Please attach these in a separate file or send them by email, we will incorporate them accordingly.
The synopsis image or visual abstract to illustrate should be uploaded as a PNG file 550 px wide x 300-600 px high.
- We would also encourage you to include the source data for figure panels that show essential data. Numerical data should be provided as individual .xls or .csv files (including a tab describing the data). For blots or microscopy, uncropped images should be submitted (using a zip archive if multiple images need to be supplied for one panel). Additional information on source data and instruction on how to label the files are available at
.
- Appendix: Please compile the tables S1 and S2 in 1 Appendix PDF file, rename them "Appendix Table S1 and S2", add the corresponding legends and a table of content.
- Please carefully check that all your figures are referenced to in the main text (callouts are currently missing for Fig. EV2D and Table S2).

As part of the EMBO Publications transparent editorial process initiative (see our Editorial at http://embomolmed.embopress.org/content/2/9/329), EMBO Molecular Medicine will publish online a Review Process File (RPF) to accompany accepted manuscripts.
This file will be published in conjunction with your paper and will include the anonymous referee reports, your point-by-point response and all pertinent correspondence relating to the manuscript. Let us know whether you agree with the publication of the RPF and as here, if you want to remove or not any figures from it prior to publication.
Please note that the Authors checklist will be published at the end of the RPF.

I look forward to receiving your revised manuscript.

With kind regards,

Lise Roth

Lise Roth, PhD
Editor
EMBO Molecular Medicine

***** Reviewer's comments *****

Referee #2 (Comments on Novelty/Model System for Author):

The strategy of boosting NK cell activity using RNAi carried by targeted nanoparticles is promising. However, as the major concerns regarding the limitations of the model were not addressed, in its present form, the manuscript is not yet suitable for publication. However, if the text were appropriately modified to avoid overstatement and more clearly describe the caveats it could be.

Referee #2 (Remarks for Author):

Main concern
In the point-by-point response reviewer figure, monocytes and granulocytes are demonstrated to take up the NPs. Even though the y axis hasn't been normalised, it is still clear that all granulocytes have high incorporation of PE-NP (even more than NK cells), and that the fluorescence of the monocytes is so high, that it seems to be off scale to the right.

Showing that in vivo KIR negative NK cells did not incorporate the NPs cannot be extrapolated to all non-NK cells, as NK cells are not phagocytes. The blood flow might help to reduce unspecific incorporation, but this was not tested.

Whereas none of this is stated in the manuscript, it is mentioned that the hyaluronan coating allows the NPs "to escape recognition and clearance by phagocytic cells", "NPs that are cell-specific may be more efficient", and the strategy has "anticipated safety". These overstatements are misleading, moreover considering that both the siRNAs and anti-NKp46 antibody tested in mice targeted human molecules.

However, if these caveats were carefully and clearly stated, and the focus kept on the potential for using RNAi to boost NK cells at the tumour site by systemic delivery of targeted nanoparticles and their impact on tumour growth and survival in this model, the report would still hold interest for the field.

Minor concerns:
There are several text/presentation issues that should be addressed for greater clarity. The list below is not exhaustive and highlights the need for more careful proof-reading.
-Page 8 "Moreover YTS-2DL1 transfected with..." is a repetition of a result already mentioned in the previous sentence
-Page 13, Figure EV2C does not show degranulation
-Last sentence of page 14 is unconnected
-There are mismatches between the n stated in the figure legends and the number of dots displayed in the plots and there is no information about the n of the measurements of intracellular calcium levels.

Referee #3 (Comments on Novelty/Model System for Author):

The authors correctly addressed all my concerns raised during the first evaluation of the manuscript. This improved the manuscript and makes me endorse it for publication.

To the Editor,

We thank the editor and reviewers for their comments. We have addressed all the points raised by the reviewers, and we hope that based on these revisions, our manuscript will now be found suitable for publication in EMBO Molecular Medicine.

Referee #2

Main concern

“In the point-by-point response reviewer figure, monocytes and granulocytes are demonstrated … The blood flow might help to reduce unspecific incorporation, but this was not tested.”

The text was modified accordingly.

“Whereas none of this is stated in the manuscript, it is mentioned that the hyaluronan coating allows the NPs "to escape recognition and clearance by phagocytic cells", "NPs that are cell-specific may be more efficient", and the strategy has "anticipated safety". These overstatements are misleading, moreover considering that both the siRNAs and anti-NKp46 antibody tested in mice targeted human molecules. However, if these caveats were carefully and clearly stated, and the focus kept on the potential for using RNAi to boost NK cells at the tumour site by systemic delivery of targeted nanoparticles and their impact on tumour growth and survival in this model, the report would still hold interest for the field.”

We have changed and modified the text according to the reviewer’s comments in order to clearly and reliably describe the technology and its future impact.

As pointed below, we have modified the sentences related to the specificity of the NPs

- “NK cell-specific NPs” was replaced in the text to “NK cell-targeting NPs” (page 10 paragraph 1 line 3)
- In the discussion the statement on page 21 line 2-3 "enabling the NPs…by phagocytic cells" was deleted.
- The second paragraph on page 21 (lines 10-23) was edited based on the reviewer's comments.
- The sentence on page 22 paragraph 2 line 12 "Thus, NPs may be… reduced side effects" was modified.
- The first paragraph on page 23 (lines 1-4) was edited based on the reviewer's comments.

<u>Minor concerns:</u>

1.”There are several text/presentation issues that should be addressed for greater clarity. The list below is not exhaustive and highlights the need for more careful proof-reading.”

All the points raised by the reviewer were addressed and corrected and the text was indeed carefully proof-read.

2.”Page 8 "Moreover YTS-2DL1 transfected with..." is a repetition of a result already mentioned in the previous sentence”

The text was changed accordingly.

3.”Page 13, Figure EV2C does not show degranulation”

We have revised the text accordingly.

4.”Last sentence of page 14 is unconnected”

The text was corrected.

5.”There are mismatches between the n stated in the figure legends and the number of dots displayed in the plots and there is no information about the n of the measurements of intracellular calcium levels.”

Thank you for bringing this to our attention. We have revised the text, updated the number of repetitions, and wrote in the missing places the number of experiments that were conducted.

31st Aug 2021

Dear Prof. Barda-Saad,

Thank you for the submission of your revised manuscript to EMBO Molecular Medicine, and please accept my apologies for the delay in getting back to you, as I was recently out of office.
We have received the enclosed report from referee #2, and as you will see, this referee is now supportive of publication. I am therefore pleased to inform you that we will be able to accept your manuscript once the following minor editorial points will be addressed:

1/ Main manuscript text:
- Please address the queries from our data editors in track changes mode in the main manuscript file labelled 'Data edited MS file'. Please use this file for any further modification (in track changes mode).
- During our standard cross-check analysis, minor text similarities with previously published materials were found (see screenshots attached), therefore please slightly modify these sentences (pages 21 and 25).
- Material and methods:
o Cells: please indicate the origin of the cells, the culture conditions and whether the cells were tested for mycoplasma contamination.
o Please provide a brief description of the Western Blot experiments. In general, when referring to previously published methods, please make sure that enough information is nevertheless provided or accessible to ensure reproducibility of the experiments.
o Animals: please provide information on age and gender.
o Patient material: include a statement that informed consent was obtained from all subjects and that the experiments conformed to the principles set out in the WMA Declaration of Helsinki and the Department of Health and Human Services Belmont Report. Please identify the committee approving the protocol.

2/ Figures and Appendix:
- The fluorescence in Fig. 2C and EV2C EV4A is difficult to see. Would you have other pictures, or could you increase the contrast to improve the staining visibility?
- In some graphs (i.e. Fig. EV1D), the individual data points are difficult to see. Could the color code or thickness of the line be modified to see these points better?

3/ Checklist:
- Please fill the author's information (top left corner)
- Please fill sections 11 and 12 on human subjects.
- Section F18: please provide a statement that you provided a Data Availability Section in the manuscript.

4/ Please note that all corresponding authors are required to supply an ORCID ID for their name upon submission of a revised manuscript. An ORCID identifier is missing for J. Benichou.

5/ Thank you for providing The Paper Explained section. The first part (Problem) needs to be shortened (approximately by half) to fit our format.

6/ Thank you for providing a synopsis text. I edited it slightly to fit our style and character count, please let me know if you agree with the following:

Natural Killer (NK) cells serve as a first line of immune defense against tumor growth and viral infections. This study demonstrates a nanobiology-based drug delivery system to enhance NK cytotoxicity by suppressing intracellular inhibitory checkpoints in the tumor microenvironment.
- NK cytotoxicity was enhanced by nano-carriers encapsulating siRNAs that target the negative regulatory genes SHP-1 and Cbls.
- Tumor growth was suppressed by these molecularly modified NK cells in-vivo.

Thank you for providing a nice synopsis picture. Please increase the size of the text to ensure readability. Please also be aware that the size of the picture should be 550 px wide x 300-600 px high.
Please also let me know if you agree with the eTOC attached (cropped from your synopsis image for online table of content).

7/ As part of the EMBO Publications transparent editorial process initiative, EMBO Molecular Medicine will publish online a Review Process File (RPF) to accompany accepted manuscripts.
This file will be published in conjunction with your paper and will include the anonymous referee reports, your point-by-point response and all pertinent correspondence relating to the manuscript. Let us know whether you agree with the publication of the

RPF and as here, IF YOU WANT TO REMOVE OR NOT ANY FIGURES from it prior to publication.
Please note that the Authors checklist will be published at the end of the RPF.

I look forward to receiving your revised manuscript.

With kind regards,

Lise Roth
Lise Roth, PhD
Editor
EMBO Molecular Medicine

***** Reviewer's comments *****

Referee #2 (Remarks for Author):

The authors have done a good job addressing the concerns from the previous review in the text of the new version

The authors performed the requested editorial changes.

30th Sep 2021

Dear Prof. Barda-Saad,

Thank you for submitting the revised files, and for bearing with the last editorial issues. We are pleased to inform you that your manuscript is accepted for publication and is now being sent to our publisher to be included in the next available issue of EMBO Molecular Medicine.

Please note that an ORCID identifier is still missing for J. Benichou, and is required before we can proceed with online publication.

Please read below for additional IMPORTANT information regarding your article, its publication and the production process.

Congratulations on your interesting work!

Lise Roth

Lise Roth, Ph.D
Editor
EMBO Molecular Medicine

Follow us on Twitter @EmboMolMed
Sign up for eTOCs at embopress.org/alertsfeeds

EMBO PRESS

YOU MUST COMPLETE ALL CELLS WITH A PINK BACKGROUND

PLEASE NOTE THAT THIS CHECKLIST WILL BE PUBLISHED ALONGSIDE YOUR PAPER

Corresponding Author Name: Mira Barda Saad	
Journal Submitted to: EMBO Molecular Medicine	
Manuscript Number: 2021-14073-V3	

Reporting Checklist For Life Sciences Articles (Rev. June 2017)

This checklist is used to ensure good reporting standards and to improve the reproducibility of published results. These guidelines are consistent with the Principles and Guidelines for Reporting Preclinical Research issued by the NIH in 2014. Please follow the journal's authorship guidelines in preparing your manuscript.

A- Figures
1. Data
The data shown in figures should satisfy the following conditions:

- the data were obtained and processed according to the field's best practice and are presented to reflect the results of the experiments in an accurate and unbiased manner.
- figure panels include only data points, measurements or observations that can be compared to each other in a scientifically meaningful way.
- graphs include clearly labeled error bars for independent experiments and sample sizes. Unless justified, error bars should not be shown for technical replicates.
- if n< 5, the individual data points from each experiment should be plotted and any statistical test employed should be justified
- Source Data should be included to report the data underlying graphs. Please follow the guidelines set out in the author ship guidelines on Data Presentation.

2. Captions

Each figure caption should contain the following information, for each panel where they are relevant:

- a specification of the experimental system investigated (eg cell line, species name).
- the assay(s) and method(s) used to carry out the reported observations and measurements
- an explicit mention of the biological and chemical entity(ies) that are being measured.
- an explicit mention of the biological and chemical entity(ies) that are altered/varied/perturbed in a controlled manner.

- the exact sample size (n) for each experimental group/condition, given as a number, not a range;
- a description of the sample collection allowing the reader to understand whether the samples represent technical or biological replicates (including how many animals, litters, cultures, etc.).
- a statement of how many times the experiment shown was independently replicated in the laboratory.
- definitions of statistical methods and measures:
  - common tests, such as t-test (please specify whether paired vs. unpaired), simple χ2 tests, Wilcoxon and Mann-Whitney tests, can be unambiguously identified by name only, but more complex techniques should be described in the methods section;
  - are tests one-sided or two-sided?
  - are there adjustments for multiple comparisons?
  - exact statistical test results, e.g., P values = x but not P values < x;
  - definition of 'center values' as median or average;
  - definition of error bars as s.d. or s.e.m.

Any descriptions too long for the figure legend should be included in the methods section and/or with the source data.

In the pink boxes below, please ensure that the answers to the following questions are reported in the manuscript itself. Every question should be answered. If the question is not relevant to your research, please write NA (non applicable). We encourage you to include a specific subsection in the methods section for statistics, reagents, animal models and human subjects.

USEFUL LINKS FOR COMPLETING THIS FORM

http://www.antibodypedia.com
http://1degreebio.org
http://www.equator-network.org/reporting-guidelines/improving-bioscience-research-repor
http://grants.nih.gov/grants/olaw/olaw.htm
http://www.mrc.ac.uk/Ourresearch/Ethicsresearchguidance/Useofanimals/index.htm
http://ClinicalTrials.gov
http://www.consort-statement.org
http://www.consort-statement.org/checklists/view/32-consort/66-title
http://www.equator-network.org/reporting-guidelines/reporting-recommendations-for-tum
http://datadryad.org
http://figshare.com
http://www.ncbi.nlm.nih.gov/gap
http://www.ebi.ac.uk/ega
http://biomodels.net/
http://biomodels.net/miriam/
http://jjj.biochem.sun.ac.za
https://osp.od.nih.gov/biosafety-biosecurity-and-emerging-biotechnology/
http://www.selectagents.gov/

B- Statistics and general methods

Please fill out these boxes (Do not worry if you cannot see all your text once you press return)

1.a. How was the sample size chosen to ensure adequate power to detect a pre-specified effect size?	No sample size was predetermined. For in-vitro assays, at least 3 independent experiments were preformed unless otherwise mentioned. For Imaging studies including IHC analysis were conducted on a large quantity of cells with the number of cells, N, listed in the figure legends or in the text.
1.b. For animal studies, include a statement about sample size estimate even if no statistical methods were used.	For in-vivo experiments, at least 12 mice were used per group.No sample size calculation was performed. Randomization was used to divide up the animals for in vivo treatment study.
2. Describe inclusion/exclusion criteria if samples or animals were excluded from the analysis. Were the criteria pre-established?	For analysis of the tumor model, only mice that developed tumors were included to guarantee comparable growth kinetcs
3. Were any steps taken to minimize the effects of subjective bias when allocating animals/samples to treatment (e.g. randomization procedure)? If yes, please describe.	Mice were stratified into random groups
For animal studies, include a statement about randomization even if no randomization was used.	Mice were stratified into random groups
4.a. Were any steps taken to minimize the effects of subjective bias during group allocation or/and when assessing results (e.g. blinding of the investigator)? If yes please describe.	The technician who operated the Mestro was blinded to group allocation.
4.b. For animal studies, include a statement about blinding even if no blinding was done	For in vivo experiments, no blinding was performed as knowledge of the experimental condition was required during treatment. For analyses of the biodistribution the technician was blinded to group allocation.
5. For every figure, are statistical tests justified as appropriate?	For single comparisons, one- and two-tailed Student's t-test or non-parametric Mann Whitney test were used. For multiple comparison, two-way ANOVA or one sample t-test followed by one way ANOVA was used .
Do the data meet the assumptions of the tests (e.g., normal distribution)? Describe any methods used to assess it.	Normality of distribution was determined by ShapiroWilk test
Is there an estimate of variation within each group of data?	As measure of variation standard error (SEM)

Is the variance similar between the groups that are being statistically compared?	Yes

C- Reagents

6. To show that antibodies were profiled for use in the system under study (assay and species), provide a citation, catalog number and/or clone number, supplementary information or reference to an antibody validation profile. e.g., Antibodypedia (see link list at top right), 1DegreeBio (see link list at top right).	For all antibodies used in this study we provided the catalogue numbers and the respective companies it was purchased from in the Appendix Data Table 2. The anibodies that were used in this study are commercially available.
7. Identify the source of cell lines and report if they were recently authenticated (e.g., by STR profiling) and tested for mycoplasma contamination.	YTS NK cell line, expressing the inhibitory receptor KIR2DL1 (YTS-KIR2DL1); two variants of the human 721.221 B-cell lymphoma line, 721.221-HLA-Cw4 and 721.221-HLA-Cw7, the human erythroleukemia, K562 were generously provided by Prof. Ofer Mandelboim (Department of Microbiology and Immunology, Faculty of Medicine, Hebrew University, Israel). 721.221-HLA-Cw4, and 721.221-HLA-Cw7 expressing stabley mCherry fluorescent protein were generated by us .

* for all hyperlinks, please see the table at the top right of the document

D- Animal Models

8. Report species, strain, gender, age of animals and genetic modification status where applicable. Please detail housing and husbandry conditions and the source of animals.	SCID/NOD mice were purchased from the Jackson Labs. 6-8 weeks old males were used.Mice were kept in ventilated cages under specific pathogen-free conditions at room temperature of 22C+/-2C, humidity of 45-65% and a light/dark cycle (12hours light / 12 hours dark).
9. For experiments involving live vertebrates, include a statement of compliance with ethical regulations and identify the committee(s) approving the experiments.	Housing and breeding of mice and experimental procedures were performed according to the guidelines of the Bar-Ilan University Ethics Committee.
10. We recommend consulting the ARRIVE guidelines (see link list at top right) (PLoS Biol. 8(6), e1000412, 2010) to ensure that other relevant aspects of animal studies are adequately reported. See author guidelines, under 'Reporting Guidelines'. See also: NIH (see link list at top right) and MRC (see link list at top right) recommendations. Please confirm compliance.	Done

E- Human Subjects

11. Identify the committee(s) approving the study protocol.	Research was performed and approved by Bar Ilan University etihics committee.
12. Include a statement confirming that informed consent was obtained from all subjects and that the experiments conformed to the principles set out in the WMA Declaration of Helsinki and the Department of Health and Human Services Belmont Report.	Blood samples from healthy donors were randomly collected and provided by Magen David Adom. No donor's identification information was provided.
13. For publication of patient photos, include a statement confirming that consent to publish was obtained.	N/A
14. Report any restrictions on the availability (and/or on the use) of human data or samples.	N/A
15. Report the clinical trial registration number (at ClinicalTrials.gov or equivalent), where applicable.	N/A
16. For phase II and III randomized controlled trials, please refer to the CONSORT flow diagram (see link list at top right) and submit the CONSORT checklist (see link list at top right) with your submission. See author guidelines, under 'Reporting Guidelines'. Please confirm you have submitted this list.	N/A
17. For tumor marker prognostic studies, we recommend that you follow the REMARK reporting guidelines (see link list at top right). See author guidelines, under 'Reporting Guidelines'. Please confirm you have followed these guidelines.	N/A

F- Data Accessibility

18: Provide a "Data Availability" section at the end of the Materials & Methods, listing the accession codes for data generated in this study and deposited in a public database (e.g. RNA-Seq data: Gene Expression Omnibus GSE39462, Proteomics data: PRIDE PXD000208 etc.) Please refer to our author guidelines for 'Data Deposition'.<br><br>Data deposition in a public repository is mandatory for:<br>a. Protein, DNA and RNA sequences<br>b. Macromolecular structures<br>c. Crystallographic data for small molecules<br>d. Functional genomics data<br>e. Proteomics and molecular interactions	Data Availability section is provided in the manuscript
19. Deposition is strongly recommended for any datasets that are central and integral to the study; please consider the journal's data policy. If no structured public repository exists for a given data type, we encourage the provision of datasets in the manuscript as a Supplementary Document (see author guidelines under 'Expanded View' or in unstructured repositories such as Dryad (see link list at top right) or Figshare (see link list at top right).	N/A
20. Access to human clinical and genomic datasets should be provided with as few restrictions as possible while respecting ethical obligations to the patients and relevant medical and legal issues. If practically possible and compatible with the individual consent agreement used in the study, such data should be deposited in one of the major public access-controlled repositories such as dbGAP (see link list at top right) or EGA (see link list at top right).	N/A
21. Computational models that are central and integral to a study should be shared without restrictions and provided in a machine-readable form. The relevant accession numbers or links should be provided. When possible, standardized format (SBML, CellML) should be used instead of scripts (e.g. MATLAB). Authors are strongly encouraged to follow the MIRIAM guidelines (see link list at top right) and deposit their model in a public database such as Biomodels (see link list at top right) or JWS Online (see link list at top right). If computer source code is provided with the paper, it should be deposited in a public repository or included in supplementary information.	N/A

G- Dual use research of concern

22. Could your study fall under dual use research restrictions? Please check biosecurity documents (see link list at top right) and list of select agents and toxins (APHIS/CDC) (see link list at top right). According to our biosecurity guidelines, provide a statement only if it could.	N/A
